# Giant virus diversity and host interactions through global metagenomics

**DOI:** 10.1038/s41586-020-1957-x

**Published:** 2020-01-22

**Authors:** Frederik Schulz, Simon Roux, David Paez-Espino, Sean Jungbluth, David A. Walsh, Vincent J. Denef, Katherine D. McMahon, Konstantinos T. Konstantinidis, Emiley A. Eloe-Fadrosh, Nikos C. Kyrpides, Tanja Woyke

**Affiliations:** 1 DOE Joint Genome Institute, Lawrence Berkeley National Laboratory, Berkeley, CA USA; 2 Groupe de recherche interuniversitaire en limnologie, Department of Biology, Concordia University, Montréal, Québec Canada; 3 Department of Ecology and Evolutionary Biology, University of Michigan, Ann Arbor, MI USA; 4 Department of Bacteriology, University of Wisconsin-Madison, Madison, WI USA; 5 Department of Civil and Environmental Engineering, University of Wisconsin-Madison, Madison, WI USA; 6 School of Civil and Environmental Engineering, Georgia Institute of Technology, Atlanta, GA USA

**Keywords:** Biodiversity, Evolution, Environmental microbiology, Viral evolution, Virus-host interactions

## Abstract

Our current knowledge about nucleocytoplasmic large DNA viruses (NCLDVs) is largely derived from viral isolates that are co-cultivated with protists and algae. Here we reconstructed 2,074 NCLDV genomes from sampling sites across the globe by building on the rapidly increasing amount of publicly available metagenome data. This led to an 11-fold increase in phylogenetic diversity and a parallel 10-fold expansion in functional diversity. Analysis of 58,023 major capsid proteins from large and giant viruses using metagenomic data revealed the global distribution patterns and cosmopolitan nature of these viruses. The discovered viral genomes encoded a wide range of proteins with putative roles in photosynthesis and diverse substrate transport processes, indicating that host reprogramming is probably a common strategy in the NCLDVs. Furthermore, inferences of horizontal gene transfer connected viral lineages to diverse eukaryotic hosts. We anticipate that the global diversity of NCLDVs that we describe here will establish giant viruses—which are associated with most major eukaryotic lineages—as important players in ecosystems across Earth’s biomes.

## Abstract (web summary)

Analysis of metagenomics data revealed that large and giant viruses are globally widely distributed and are associated with most major eukaryotic lineages.

## Main

Large and giant viruses of the NCLDV supergroup have complex genomes with sizes of up to several megabases, and virions that are a similar size to, or even larger than, small cellular organisms^1–3^. These viruses infect a wide range of eukaryotes from protists to animals^4^. Marker gene surveys have shown that NCLDVs are not only extremely abundant and diverse in oceans^5–7^, but can also frequently be found in freshwater^8^ and soil^9^. However, the discovery of large and giant viruses has mainly been driven by their co-cultivation with amoebae or isolation together with their native hosts^1,4,8^. Only recently, metagenomic and single-cell genomic studies have facilitated the discovery of several new NCLDV members and showed that cultivation-independent methods are applicable to these viruses just as they are to uncultivated Bacteria and Archaea^9–14^.

**Extended Data Fig. 1:**
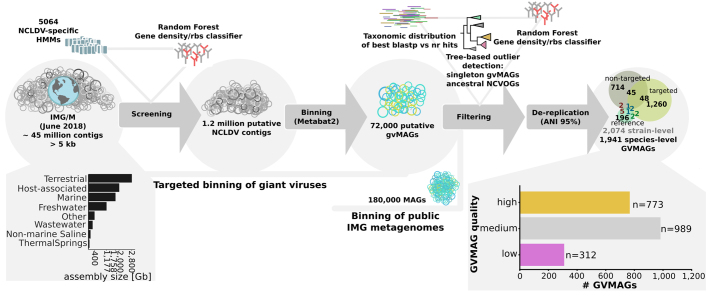
Discovery pipeline for GVMAGs. Approximately 46 million contigs that were longer than 5 kb and were available in IMG/M^15^ (June 2018) were screened for potential NCLDV contigs using a combination of 5,064 NCLDV-specific HMMs and a random-forest classifier based on gene density and RBS motifs. The resulting set of 1.2 million contigs was then subjected to metagenomic binning using MetaBAT2^53^, with binning performed separately for each metagenome that contained putative NCLDV contigs. To the resulting approximately 72,000 GVMAGs, we added around 180,000 low-quality MAGs based on MIMAG^55^ that were generated by non-targeted binning of metagenomes in IMG/M. The resulting set of approximately 252,000 GVMAGs and MAGs were then filtered on the basis of assembly size and using a combination of the consensus of taxonomic affiliation of best blast hits across contigs, the presence or absence and copy numbers of frequently conserved NCLDV genes taking into account neighbouring taxa in the species tree and random-forest classifier based on gene density and RBS motifs. Outlier contigs were removed as described in the Methods and only MAGs that showed a copy-number distribution of frequently conserved NCLDV genes similar to closely related viral genomes were maintained in the final dataset.

**Extended Data Fig. 2:**
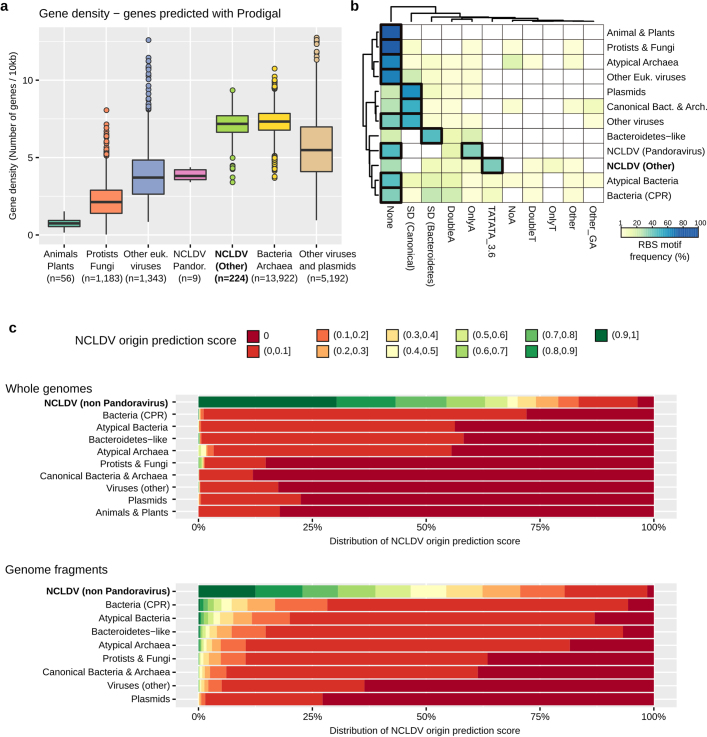
The RBS classifier. Unique features of NCLDV genomes and efficiency of random-forest classifiers based on these features. **a**, Gene density (*y* axis, average number of genes predicted per 10 kb of genome) for genomic sequences from different types of organisms or entities (*x* axis). Genomes were grouped on the basis of taxonomy (kingdom and domain ranks) as well as patterns of RBS motifs and gene density. ‘Other euk. viruses’, non-NCLDV eukaryotic viruses; ‘NCLDV Pandor.’, pandoravirus and similar NCLDVs; ‘NCLDV (Other)’, non-pandoravirus NCLDVs. Centre lines of box plots represent the median, bounds of the boxes indicate the lower and upper quartiles, whiskers extend to points that lie within 1.5× the interquartile range of the lower and upper quartiles. Sample sizes (number of genomes) are indicated. **b**, Frequency of RBS motifs identified across different genomes groups. RBS motif frequencies were based on prodigal gene prediction using the ‘full motif scan’ option. For clarity, only RBS motif frequencies >1% are displayed. RBS motif frequencies ≥30% are highlighted with a bold outline. ‘Other Euk. viruses’, non-NCLDV eukaryotic viruses; ‘NCLDV (pandoravirus)’, pandoravirus and similar NCLDVs; ‘NCLDV (Other)’, non-pandoravirus NCLDVs. **c**, Predictions of NCLDV origin on the basis of genome features and predicted RBS motifs by random-forest classifiers for complete genomes (top) and short genome fragments (bottom). Predictions for individual genomes were obtained through a tenfold cross-validation. Similar results were obtained when predicting only two classes (NCLDV and non-NCLDV, displayed here) or when predicting classes corresponding to the eight types of genomes. CPR, candidate phyla radiation; SD, Shine–Dalgarno sequence.

**Extended Data Fig. 3:**
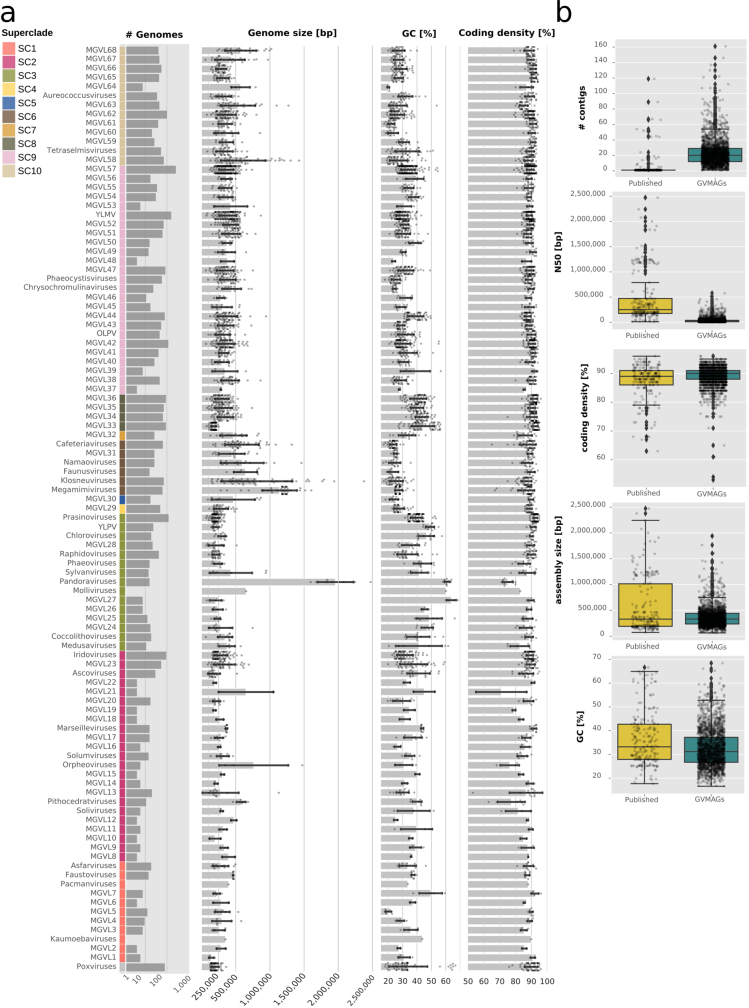
Features of GVMAGs. **a**, Mean assembly size, GC content and coding density for each lineage in the NCLDV, coloured by superclade, individual data points are shown. Data are mean ± s.d. **b**, Assembly metrics of all GVMAGs compared to previously published NCLDV genomes included in this study. Centre lines of box plots represent the median, bounds of boxes indicate the lower and upper quartiles, whiskers extend to points that lie within 1.5× interquartile range of the lower and upper quartiles. Sample size for the published data is 205 genomes and for GVMAGs is 2,074 genomes.

**Extended Data Fig. 4:**
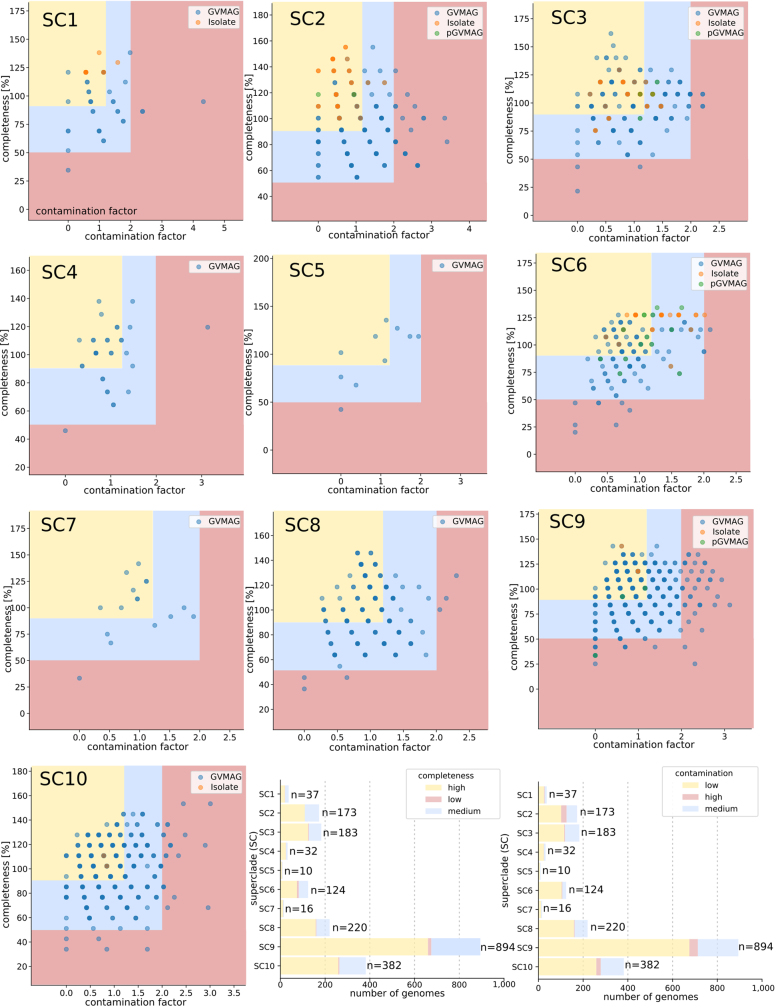
Estimated completeness and contamination of GVMAGs on the basis of the presence of conserved NCVOGs. Scatter plots show estimated completeness and contamination for GVMAGs in each superclade (SC), previously published GVMAGs (pGVMAGs) and isolate genomes (filled circles with different colours) compared with the average of the respective superclade. Genomes in the red area were classified as low quality, genomes in the blue area were classified as medium quality and genomes in the yellow area were classified as high quality on the basis of the combination of completeness and contamination. Stacked bars (bottom right) summarize, for each NCLDV superclade, the total number of GVMAGs with low, medium and high contamination and completeness.

Here, we have used a multistep metagenome data-mining, binning and iterative-filtering pipeline (Extended Data Figs. 1, 2 and Supplementary Text 1), which led to the recovery of genomes representing 2,074 putative NCLDV populations from 8,535 publicly available metagenomes in the Integrated Microbial Genomes and Microbiomes (IMG/M) database^15^. The assembly size, GC content, coding density and copy number of nucleocytoplasmic virus orthologous genes (NCVOGs)^16^ were comparable to previously described NCLDV genomes, supporting the classification of these genomes as giant virus metagenome-assembled genomes (GVMAGs) (Extended Data Figs. 3, 4 and Supplementary Tables 1–3). Using an approach that relied on conserved NCVOGs, we estimated genome completeness and contamination, which led to the classification of 773 high-quality, 989 medium-quality and 312 low-quality GVMAGs (Extended Data Figs. 1, 4 and Supplementary Tables 1, 4), in line with the MIUViG recommendations^17^.

**Fig. 1:**
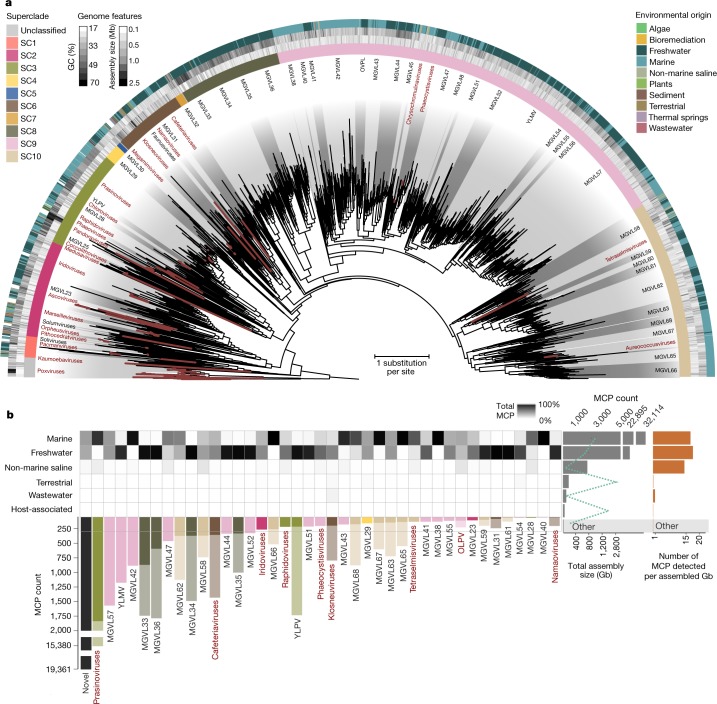
Metagenomic expansion of the NCLDV diversity. **a**, Maximum-likelihood phylogenetic tree of the NCLDV inferred from a concatenated protein alignment of five core NCVOGs^16^. Branches in dark red represent published genomes and branches in black represent GVMAGs generated in this study. Shades of grey indicate boundaries of genus- and subfamily-level clades; previously described lineages are labelled. Tree annotations from inside to the outside: (1) superclade (SC), (2) GC content, (3) assembly size and (4) environmental origin. **b**, Distribution of NCLDV lineages across different habitats. The bars adjacent to the heat map show the total number of detected MCPs per habitat (facing to the right) and per lineage (facing downwards) as total count (total bar length) and corrected count on the basis of the average copy number of MCPs in the respective lineage (darker shaded bar length). The plot includes only lineages for which at least 100 MCPs could be detected. NCLDV lineages with available virus isolates are indicated in red. The turquoise dashed line indicates the total size of the metagenome assemblies that were screened in this analysis. Bars on the far right indicate, for each environment, the number of detected MCPs per assembled gigabase (Gb).

**Extended Data Fig. 5:**
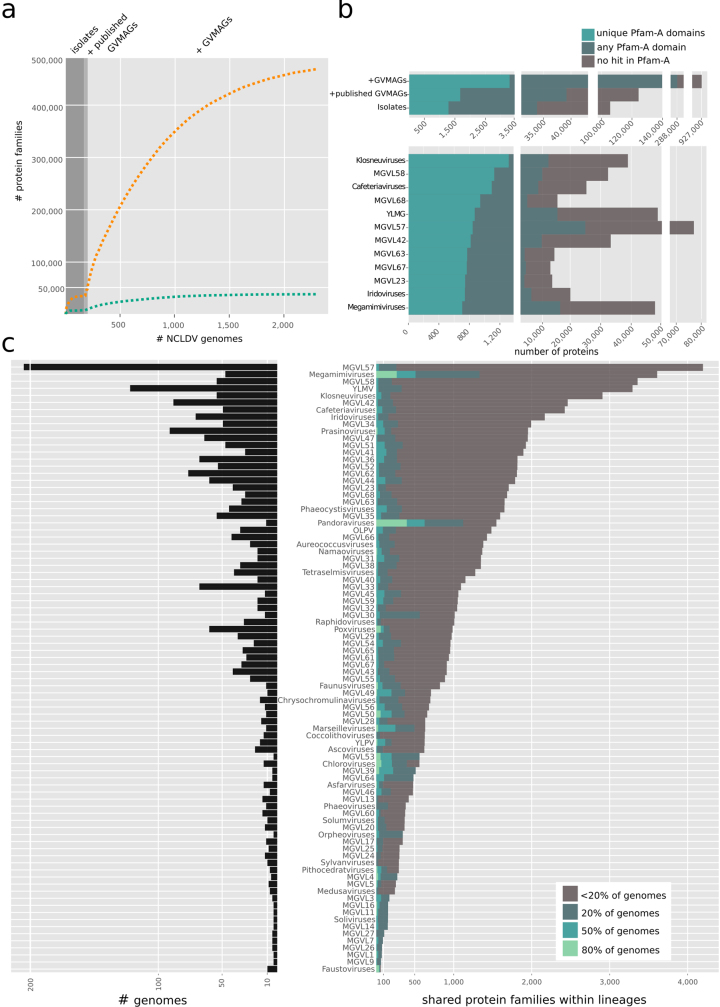
Shared and unique protein families within NCLDV lineages. **a**, Collectors curve showing the increase in functional diversity estimated on the basis of the total number of protein families detected in NCLDV isolates, previously published GVMAGs and GVMAGs recovered in this study. The orange curve includes all detected protein families; the blue curve only includes protein families that included by at least two proteins. **b**, Top, the total number of different Pfam-A domains, total number of proteins with any Pfam-A domain and total number of proteins found in NCLDV isolates, previously published NCLDV genomes from metagenomes and GVMAGs recovered in this study. Bottom, NCLDV lineages with the greatest number of unique Pfam-A domains. **c**, The total number of genomes per lineage (left) and total number of protein families (at least two members) found in each lineage are indicated together with the proportion of genomes in the respective lineage that share protein families (right).

**Extended Data Fig. 6:**
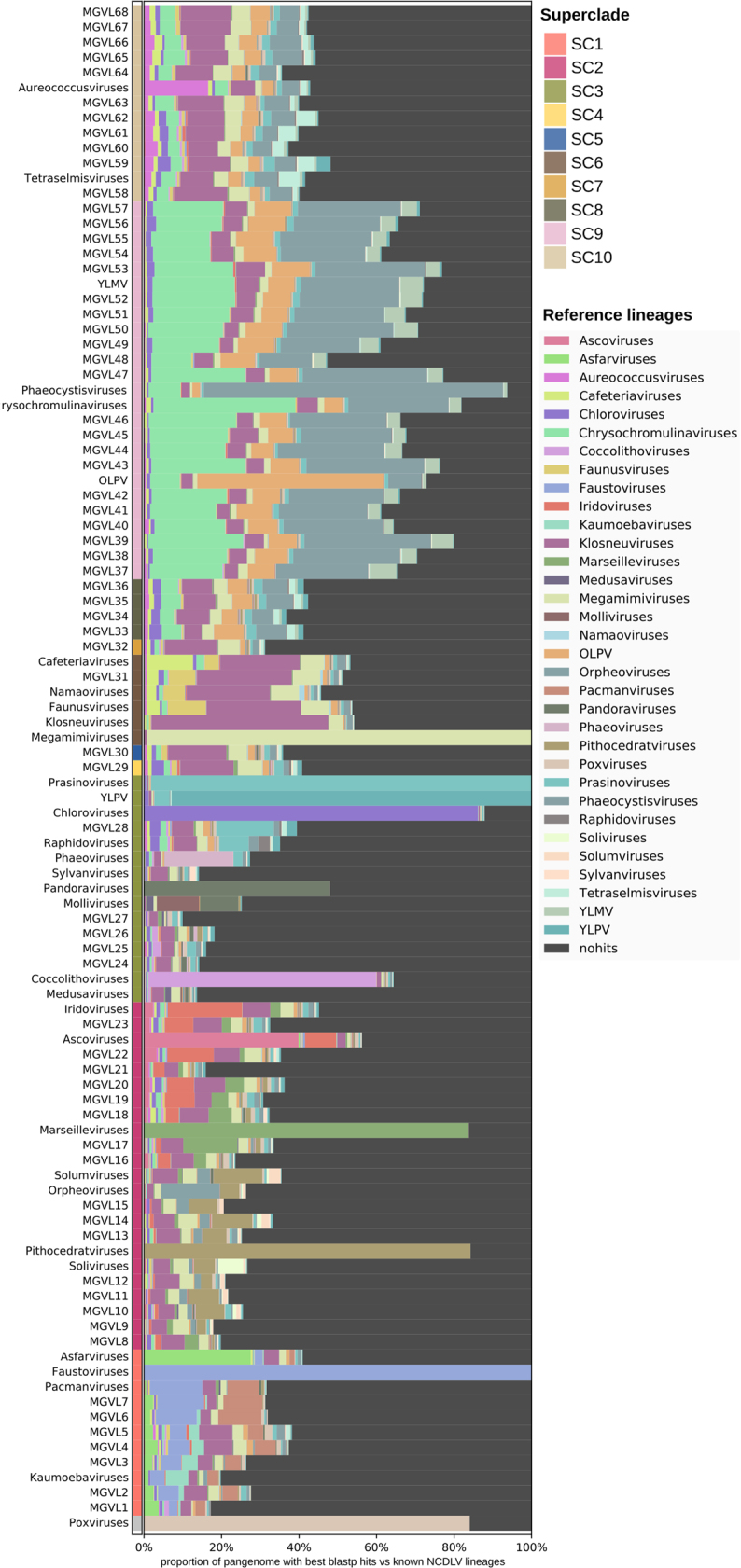
Similarity of proteins encoded in expanded NCLDV lineages and new MGVLs to known NCLDV proteins. For each lineage the proportion of encoded proteins with homology (*E*-value cut-off of 1 × 10^−5^) to known NCLDV proteins is shown.

Augmenting the existing NCLDV phylogenetic framework with the GVMAGs substantially increased the diversity of this proposed viral order (Fig. 1a and Supplementary Data 1). The resulting phylogenetic tree expanded from 205 to 2,279 viral genomes, which can now be divided into 100 potentially genus- or subfamily-level monophyletic clades spanning 10 provisional superclades, compared with the previously recognized 20 genera^2^. This translates into an 11-fold increase in phylogenetic diversity of the NCLDVs. Notably, the addition of the novel viral genomes did not change the basic topology of the NCLDV tree but rather altered the contribution of existing groups, the *Mimiviridae* in particular, to the total viral diversity. Furthermore, the presence of conserved NCVOGs in lineage-specific patterns strengthens the hypothesis of a common evolutionary origin of this viral group^2^. Novel groups of viruses with no isolate representatives appeared within the existing taxonomic framework (that is, metagenomic giant virus lineages (MGVLs)). The greatest number of GVMAGs could be attributed to MGVL57 (*n* = 205), the Yellowstone Lake mimiviruses (YLMVs; *n* = 119) and MGVL42 (*n* = 84). In addition, several established viral lineages were considerably extended, such as the prasinoviruses (*n* = 77), iridoviruses (*n* = 59), cafeteriaviruses (*n* = 43), phaeocystisviruses (*n* = 37), klosneuviruses (*n* = 36), tetraselmisviruses (*n* = 34) and raphidoviruses (*n* = 26), some of which previously consisted of single isolates. In total, the GVMAGs increased the 123,000 previously known NCLDV proteins that clustered in 47,700 protein families to more than 924,000 proteins in 508,000 protein families (Extended Data Fig. 5a). Pfam-A protein domains could be assigned to less than one third (31%) of these proteins (Extended Data Fig. 5b). The potentially most-versatile viral lineage on the basis of known gene functions were the klosneuviruses, for which more than 1,200 different protein domains could be detected (Extended Data Fig. 5b). MGVL57, MGVL58, YLMVs and klosneuviruses were the most-diverse lineages on the basis of their overall gene content, as indicated by a low number of shared protein families compared with the total number of protein families (Extended Data Fig. 5c). MGVL27, medusaviruses, sylvanviruses and MGVL24 represented the viral lineages with the highest genome novelty; for these lineages, on average, less than 15% of proteins showed similarity to known NCLDV proteins (Extended Data Fig. 6). Notably, clades that had been predominantly sampled in the past with several viral isolate genomes sequenced, such as marseilleviruses, poxviruses, pandoraviruses and faustoviruses, were nearly absent in the environmental microbiome data. This finding indicates that these viruses or their hosts have comparably low abundances in the samples analysed our dataset. It also suggests that there is a skew in the isolation and co-cultivation efforts of giant viruses using selected non-native hosts in laboratory setups^18–20^. Large-scale, cultivation-independent genome-resolved metagenomics alleviates such bias and provides a more-global snapshot of diversity and the spatial distribution of NCLDVs in their natural habitats.

**Extended Data Fig. 7:**
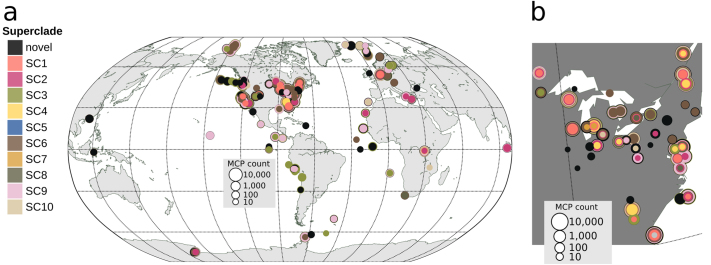
Distribution of NCLDV MCPs. **a**, Global distribution of NCLDV MCPs. **b**, A detailed view of the Midwest and East Coast of the United States and Canada. Filled circles are coloured on the basis of the affiliation with superclade and the circle diameter correlates with the number of MCPs detected at the respective sampling location. Circles at the same coordinates are stacked by size with the largest circles at the bottom. The category ‘novel’ contains all MCPs that could not be assigned to any of the superclades.

To further deepen our understanding of the environmental distribution patterns of the NCLDVs, we performed a survey of the major capsid protein (MCP) across all public metagenomic datasets. We identified more than 58,000 copies of this protein, of which 67% could be assigned to viral lineages (Fig. 1b). Among the most-commonly found lineages were prasinoviruses, MGVL57 and YLMV with more than 1,000 occurrences each. At the same time, only a few MCPs (less than 100) were detected in viruses that have repeatedly been isolated in co-cultivation with amoebae, such as megamimiviruses, marseilleviruses and faustoviruses^18–20^. In our environmental survey, MCPs were predominantly found in marine (around 55%) and freshwater (about 40%) and—to a much lesser extent—in terrestrial (less than 1%) environments. Some NCLDV lineages occurred solely in either freshwater (YLMV, MGVL33 and MGVL36) or marine (prasinoviruses, MGVL42 and MGVL66) systems, whereas members of other lineages were found in both—or in an even-wider range of—environments (such as klosneuviruses, which were found in freshwater, marine, non-marine saline, terrestrial, wastewater and host-associated ecosystems). Large and giant viruses could also be detected in hydrothermal vents and thermal springs; however, comparably few MCPs were present in these habitats (Fig. 1b). Projecting the distribution of NCLDVs onto a global scale makes their ubiquitous nature apparent (Extended Data Fig. 7). These viruses can be found almost anywhere with many different lineages often co-occurring in close proximity to each other, suggesting that their discovery is chiefly limited by sampling effort.

**Fig. 2:**
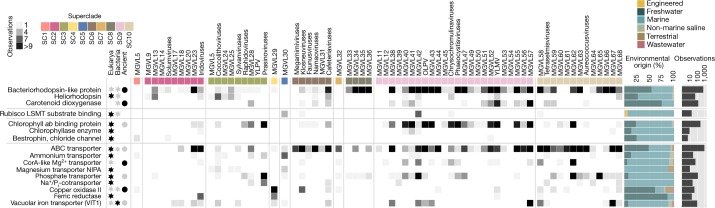
NCLDV coding potential and proteins that are probably involved in metabolic host reprogramming. Copy numbers of selected Pfam domains with potential roles as light-driven proton pumps, in carbon fixation, in photosynthesis and in diverse substrate transport processes. Filled stars and circles specify observed modes of transmission of the respective Pfam-domain-containing proteins. Stars represent recent HGTs from either eukaryotes or bacteria; circles indicate vertical transmission after ancient HGT or gene birth in the NCLDV; a darker colour indicates the predominantly observed mode of transmission (five or more events). The stacked bars on the right side of the heat map show, for each observed protein domain, the proportional distribution across different habitat types. Bars on the far right indicate the total number of observations for each protein domain.

Considering the ubiquitous prevalence of large and giant viruses, we aimed to investigate the potential influences that these viruses have on their hosts. The detrimental effect of viral infections on their eukaryotic hosts are well-known^1^; however, a few recent studies have shown that NCLDVs might also complement the metabolism of their host, for example, by encoding transporters that take up nutrients, such as nitrogen, or fermentation genes^21,22^. Expanding these initial findings, our data showed that diverse lineages across all NCLDV superclades encoded enzymes with potential roles in photosynthesis, diverse substrate transport processes, light-driven proton pumps and retinal pigments (Fig. 2). Maps of the presence, absence and prevalence of these genes revealed lineage- and environment-specific patterns. Most-commonly observed across a wide-range of habitats were ABC transporters, chlorophyll *ab*-binding proteins and bacteriorhodopsin-like proteins (Fig. 2, Supplementary Note 2 and Supplementary Table 5). Transporters for ammonium, magnesium and phosphate, which are likely to be of importance for hosts in oligotrophic environments such as the surface ocean, were predominantly found in marine viruses. Enzymes such as ferric reductases and multicopper oxidases—which facilitate the uptake of iron^23,24^, an essential trace element that is often growth-limiting, especially in photosynthetic organisms^25^—were encoded in GVMAGs sampled across different habitats. This wealth of virus-encoded genes with roles in energy generation and nutrient acquisition has far-reaching implications for ecosystem dynamics. Metabolic reprogramming refers to a common phenomenon in which bacterial viruses obtain genes from their hosts and maintain them to support host metabolism^26^. Our results illustrate that in a similar manner, NCLDV-mediated host reprogramming is probably an important strategy to increase viral fecundity and at the same time render a short-term competitive advantage of infected eukaryotic host cells, especially under nutrient-limited conditions.

**Extended Data Fig. 8:**
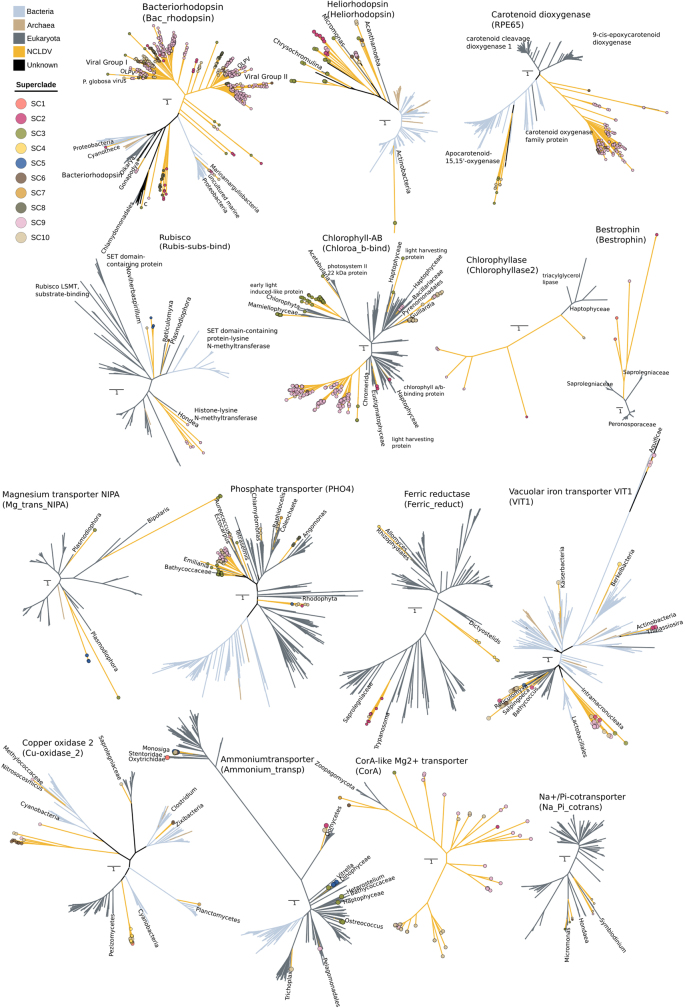
Maximum-likelihood phylogenetic trees. Maximum-likelihood phylogenetic trees that underlie the analysis in Fig. 2. Trees were inferred using IQ-tree with the following models: Na^+^/P_i_ cotranporter, LG4M + R7; ammonium transporter, LG4M + R10; bacteriorhodopsin, LG + F + R10; bestrophin, LG4M + R5; carotenoid dioxygenase, LG + F + R10; Chlorophyll *ab*, LG4M + F + R10; chlorophyllase, LG + I + G4; CorA-like Mg^2+^ transporter, LG + F + R3; copper oxidase II, LG4M + R10; heliorhodopsin, LG4M + R9; magnesium transporter NIPA, LG4M + R6; ferric reductase, LG + F + R9; phosphate transporter, LG4M + R10; Rubisco, LG4M + R6; and vacuolar iron transporter (VIT1), LG4M + R10.

**Extended Data Fig. 9:**
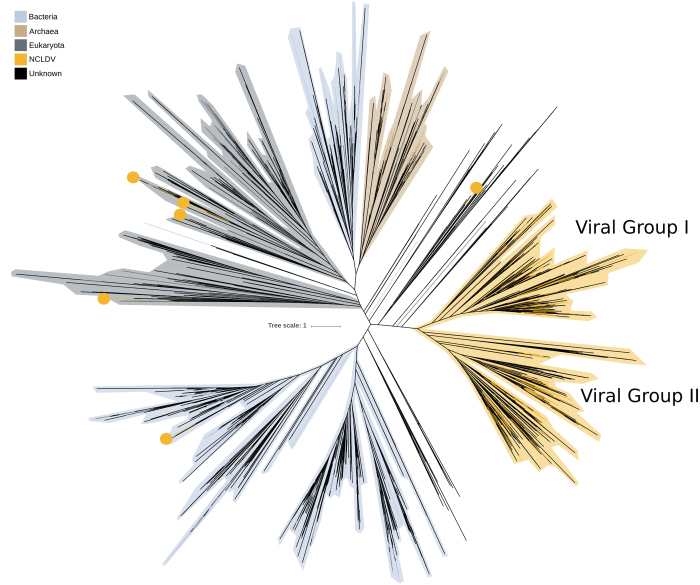
Diversity of metagenomic rhodopsins. Maximum-likelihood tree (IQ-tree, LG4M + R10 substitution model) of rhodopsins after dereplication through clustering with CD-hit at a 70% similarity threshold. Clades that predominantly include rhodopsins of archaeal, bacterial, eukaryotic or NCLDV origin are highlighted in the different colours. Yellow filled circles indicate NCLDV rhodopsins that have probably been acquired from cellular organisms through HGT.

In agreement with previous studies^27–30^, many of the identified viral genes with predicted effects on host cell processes were probably acquired from their hosts through horizontal gene transfer (HGT) (Fig. 2 and Extended Data Fig. 8). Other genes were present across different viral lineages and superclades, suggesting ancient transfer followed by vertical inheritance during the course of NCLDV evolution or the origin of the respective gene in a common ancestor of this group of viruses. A notable example is the group of rhodopsin-like domain-containing proteins, which we found in 555 of the GVMAGs. Type-1 rhodopsins in algae-infecting phycodnaviruses and in viruses of heterotrophic choanoflagellates have been reported in previous studies and comprise viral rhodopsin groups I and II^10,31,32^. However, in light of our extended sampling of NCLDV genomes, it becomes evident that NCLDVs encoded more-diverse rhodopsins than described (Extended Data Fig. 8), which comprise approximately one quarter of the total known diversity of rhodopsins and include proteins from all publicly available metagenomes (Extended Data Fig. 9). Notably, the phylogeny of the viral rhodopsins from all NCLDV superclades exhibits a strongly supported monophyletic signal, which implies that this gene might represent an ancestral trait of the NCLDV that was subsequently lost in some lineages. In addition to viral rhodopsin group I and II, additional NCLDV rhodopsins branch closely to their cellular counterparts and have probably been acquired by HGT from different hosts (Extended Data Fig. 8). In a similar manner, putative NCLDV heliorhodopsins were found intertwined with their homologues in the algae *Chrysochromulina* and *Micromonas* (Extended Data Fig. 8). In addition to the rhodopsins, our dataset contained 119 GVMAGs that encoded carotenoid oxygenases, which potentially modulate light-harvesting capacity or synthesize bioactive compounds^33^. It is conceivable that some of the NCLDV rhodopsins function in conjunction with the carotenoid oxygenases and have important roles in modulating host-cell processes; for example, by acting as light-driven proton pumps, as photoreceptors in host phototactic motility or as photoprotectants^10,34,35^—each of these functions lead to metabolic advantages of infected populations.

**Fig. 3:**
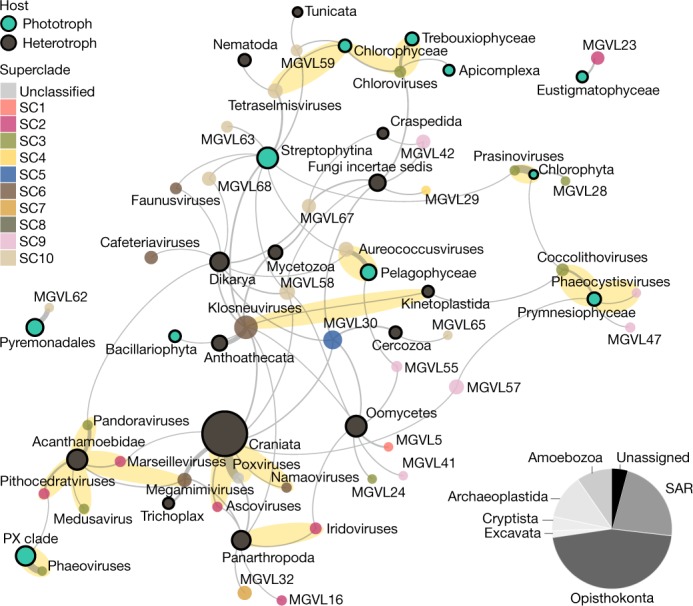
HGT between NCLDV and their putative eukaryotic hosts. Undirected HGT network with nodes that represent previously described viral lineages and MGVLs, coloured on the basis of NCLDV superclade affiliation, with names above the node and their putative hosts (highlighted in black with names below the node, coloured on the basis of lifestyle); edges are weighted on the basis of the number of detected transfers. Connections comprising at least four transfers are shown. Experimentally verified virus–host associations are highlighted in yellow with names in bold. The proportion of HGT candidates assigned to hosts from different major eukaryotic lineages is shown as a pie chart.

Uptake of host genes is a common mechanism in the evolution of NCLDVs^2,11,30,36^. Using HGT analyses, we assigned putative hosts to different NCLDV lineages. Analysis of 2,040 genes that have probably undergone HGT provided linkage information for 50 viral lineages to 32 groups of putative eukaryotic hosts (Fig. 3 and Supplementary Table 6). Notably, 17 out of 23 viral lineages that contained genomes from isolated viruses could be connected through HGT to their experimentally verified native hosts, such as most algae-infecting viruses and metazoa-infecting ascoviruses, namaoviruses and poxviruses, as well as connecting klosneuviruses to Kinetoplastida^37,38^. Our analysis further confirmed *Acanthamoeba* as a host of pandoraviruses, pithocedratviruses, medusaviruses, marseilleviruses and megamimiviruses. Notably, megamimiviruses, which have exclusively been obtained through co-cultivation with amoebae, showed not only HGT with this host but were linked even more strongly to multicellular animals. The best-connected NCLDV lineage was the klosneuviruses, a viral subfamily mainly known from metagenomic studies^9,11,12,39^. Our HGT network revealed that klosneuviruses have a diverse putative host range of mainly heterotrophs, including Anthoathecata—to which it showed the strongest connection—as well as fungi and arthropods, and different protists, including slime moulds. By contrast, Oomycetes, Dikarya, fungi incertae sedis and Streptophytina emerged as putative hosts for the greatest number of different NCLDV lineages, despite the lack of isolation of NCLDVs from any of these organisms. With predicted hosts in Opisthokonta, Amoebozoa, Excavata, Archaeoplastida, Cryptista and the Stramenopila, Alveolata, Rhizaria (SAR) supergroup, our results suggest that members of the NCLDV might be able to infect most major eukaryotic lineages^40^ (Fig. 3). This is consistent with previous reports based on eukaryotic genome data^27^ and experimental data showing that large and giant viruses infect marine arrow worms^41^, epithelial cells in fish gills^38^ and potentially also corals and sponges^42^. Of note, our analysis did not reveal linkage to human hosts. We expect that with improved sampling of host genomes—particularly genomes of underexplored protists and algae—host linkage through HGT will yield an even more comprehensive picture of the host range and evolutionary histories of NCLDVs.

Overall, we leveraged the availability of metagenomic data generated by the global sampling efforts of a community of scientists to expand our insights into the diversity, host metabolic complementation and putative host range of large and giant viruses. NCLDV infections probably occur in all major eukaryotic lineages, with repercussions for many of Earth’s major biogeochemical processes. Our data and findings represent a solid foundation and expansive resource for future giant-virus research efforts to deepen our understanding of the evolutionary and ecological bearings of these viral giants.

## Methods

### Generation of models to detect NCLDV proteins

Initial hidden Markov models (HMMs) for the MCPs were built from a multiple sequence alignment of published NCLDV MCPs and subsequently updated on the basis of extracted metagenomic NCLDV MCP sequences. We screened around 537 million proteins encoded on about 45.1 million contigs with a length greater than 5 kb available in 8,535 public metagenomes in IMG/M^43^ (June 2018) for contigs that encode the NCLDV MCP using a version of hmmsearch (v.3.1b2, http://hmmer.org/) that is optimized^44^ for the supercomputer Cori, with a set of models for the NCLDV MCP (https://bitbucket.org/berkeleylab/mtg-gv-exp/) and an *E*-value cut-off of 1 × 10^−10^. The 1,003,222 proteins found on the 77,701 contigs with hits for MCPs were then clustered with CD-hit^45^ at a sequence similarity of 99% to remove nearly identical and identical proteins. This resulted in 524,161 clusters and singletons. The cluster representatives were used to infer protein families using orthofinder (v.2.27) with default settings and the -diamond flag^46,47^. Multiple sequence alignments were built with mafft^48^ (v.7.294b) for protein families that included at least 10 members and corresponding HMM models were obtained with hmmbuild (v.3.1b2, http://hmmer.org/). This led to a total of 7,182 HMMs that can detect NCDLV proteins that were then tested against all public genomes in IMG/M^43^ (June 2018). Models that gave rise to hits above an *E*-value cut-off of 1 × 10^−10^ in more than 10 reference genomes were removed. The resulting 5,064 models were then used for targeted binning of NCLDV metagenome contigs.

### Identification of NCLDV-specific genome features and design of an automatic classifier

A set of representative genomes of bacteria, archaea, eukaryotes and non-NCLDV viruses was gathered from the IMG/M database^43^ (June 2018) and combined with NCLDV genomes assembled from metagenomes and protist genomes downloaded from NCBI GenBank to identify NCLDV-specific genome features. Genes were predicted for these genomes using Prodigal^49^ (v.2.6.3; February, 2016) in both ‘regular’ mode (default parameters) and with the option ‘-n’ activated, which forces a full motif scan. For genomes of less than 100 kb, the option ‘-p meta’ was used to apply precalculated training files rather than training the gene predictor from the genome, as recommended by the tool documentation. Next, a set of different metrics was calculated for each genome on the basis of the genes predicted with a confidence of ≥90 and score of ≥50. These included gene density (number of genes predicted on average per 10 kb of genome), coding density (number of bp predicted as part of a coding sequence per 10 kb of genome), spacer length (average length of the spacer between the predicted ribosomal binding site (RBS)), predicted start codon for genes in which a putative RBS was detected and RBS motif profile (the proportion of each type of RBS predicted in the genome, see below).

For the RBS motif profile, motifs were predicted using the full motif scan option of prodigal (see above). Notably, some of these motifs may not represent true RBSs, but are instead other conserved motifs (including transcription-related motifs) found upstream of start codons in these different genomes. These motifs were grouped into 11 categories as follows: (1) ‘None’ for cases in which prodigal did not predict a RBS; (2) ‘SD_Canonical’ for different variations of the canonical AGGAGG Shine–Dalgarno sequence (for example, AGGAG, AGxAG, GAGGA, as well as motifs identified by Prodigal as ‘3Base_5BMM’ or ‘4Base_6BMM’); (3) ‘SD_Bacteroidetes’ for variations of the motif predicted typically from Bacteroidetes genomes (TA{2,5}T{0,1}: T followed by 2–5 As, and with sometimes a terminal T); (4) ‘Other_GA’ for motifs that include ‘GA’ patterns but that are different from the canonical Shine–Dalgarno sequence, for example, GAGGGA, typically identified in a few archaeal and bacterial genomes; (5) ‘TATATA_3.6’ for variations of the motif typically detected in NCLDV, that is, a motif of 3–6 bp with alternating Ts and As (TAT, ATAT, TATA, TATAT, and so on); (6) ‘OnlyA’ for motifs exclusively composed of As not already included in a previous group, for example, AAAAA, most often found in Bacteroidetes; (7) ‘OnlyT’ for motifs exclusively composed of Ts not already included in a previous group, for example, TTTTT, found at a low frequency in some archaeal genomes; (8) ‘DoubleA’ for motifs with two consecutive As not already included in a previous group, for example, AAAAC, most often found in Bacteroidetes and bacteria from the candidate phyla radiation (CPR) group; (9) ‘DoubleT’ for motifs with two consecutive Ts not already included in a previous group, for example, TACTT, found at a low frequency in plants, Bacteroidetes and NCLDV; (10) ‘NoA’ for motifs without any As and not included in a previous group, for example, TCTCG, found in some archaeal genomes; and (11) ‘Other’ for motifs that did not fit into any of these categories.

Representative genomes were then grouped on the basis of the frequency of each motif type through hierarchical clustering (R function ‘hclust’). This enabled the delineation of 12 genome groups on the basis of taxonomy (at the kingdom or domain ranks) and motif profile (Extended Data Fig. 2). Two types of random-forest classifiers were then built on the basis of the 14 features (11 motifs, gene density, coding density and average spacer length, see above): one for which the category to be predicted was binary (that is, ‘Virus_NCLDV’ versus ‘Other’) and one for which the category to be predicted was the set of genome groups based on predicted RBS motifs (‘NCLDV (non-pandoraviruses)’, ‘animal and plants’, ‘protists & fungi’, ‘canonical bacteria and archaea’, ‘bacteroidetes-like’, ‘bacteria (CPR)’, ‘atypical bacteria’, ‘atypical archaea’, ‘plasmids’ and ‘other viruses’, which include pandoraviruses). The 14 features were evaluated on the whole genomes, as well as on fragments of 20 kb and 10 kb selected randomly along the genomes. These random fragments were used to train a classifier on input sequences more comparable to metagenome assemblies, which most often represent short genome fragments of a few kb. For these fragments, Prodigal was run with the ‘-p meta’ option and default parameters otherwise^50^, that is, without a full motif scan, as these sequences are typically too short to identify de novo RBS motifs. Animal and plant genomes were not included in this analysis as these are highly unlikely to be assembled from metagenomes. All classifiers were built using R library randomforest and included 2,000 trees, with default parameters otherwise, and 10-fold cross-validation was performed to evaluate the classifier accuracy. The probability ‘prob’ of NCLDV origin was used as a prediction score to evaluate the classifiers and was then applied to metagenome assemblies. Because the input dataset is easily skewed towards bacterial and archaeal genomes, specificity and sensitivity were evaluated separately for each group of genome (Extended Data Fig. 2c). Statistical tests were performed in R using the package stats (Kolmogorov–Smirnov test)^51^ and effsize (Cohen’s effect size)^52^.

### MAGs from non-targeted binning of IMG genomes

Complementary to the targeted binning of NCLDV contigs, we performed genome binning of public metagenomes in IMG/M (assessed June 2018)^15^ with MetaBAT (v.0.32.4)^53^ in the ‘superspecific’ mode, using read coverage information, if available in IMG, and a minimum contig length of 5 kb. Resulting MAGs were then checked for quality using CheckM (v.1.0.7)^54^. Genome bins with completeness <50% were labelled as low quality according to the ‘minimum information for a MAG’ (MIMAG) standards^55^.

### Targeted binning of putative NCLDV metagenome contigs

The 5,064 NCLDV-specific models were used for hmmsearch (v.3.1b2, http://hmmer.org/) on the initial set of around 537 million proteins encoded on about 45 million contigs with a length greater than 5 kb with an *E*-value cut-off of 1 × 10^−10^ (Extended Data Fig. 1). In addition to the screening of the metagenomic contigs with NCLDV-specific models, we also used an automatic classifier using gene density and RBS motifs (see above). On the basis of the output of the automatic classifier, a score was assigned to each contig: a score of 2 if Ratio_TATATA_36 > 0.3 or Pred_simple_NCLDV_score > 0.3 and the prediction result was ‘Virus_NCLDV’, a score of 1 if Ratio_TATATA_36 > 0.3 or Pred_simple_NCLDV_score > 0.1 or the prediction result was ‘Virus_NCLDV’, otherwise a score of 0. On the basis of the cross-validation of the classifier, these parameters were chosen to maximize sensitivity while retaining enough specificity. The resulting set of around 1.2 million contigs with an RBS score of at least 1 and/or at least 20% of encoded genes (1 out of 5) with hits to the NCLDV models were subject to metagenomic binning as follows: for each metagenome, putative NCLDV contigs were extracted and binning performed with MetaBAT^56^ (v.2) and contig read coverage information was used as input in case it was available in IMG^43^. The targeted binning approach gave rise to around 72,000 putative NCLDV MAGs.

### Filtering of GVMAGs

Contigs with a length of less than 5 kb were removed from GVMAGs. Filtering was performed on the basis of the copy number of NCVOGs^16^ (Supplementary Tables 2, 3). GVMAGs were removed when they encoded more than 20 copies of NCVOG0023, 4 copies of NCVOG0038, 12 copies of NCVOG0076, 7 copies of NCVOG0249 or 4 copies of NCVOG0262. On the basis of the copy numbers of 16 conserved NCOVGs (NCVOG0035, NCVOG0036, NCVOG0038, NCVOG0052, NCVOG0059, NCVOG0211, NCVOG0249, NCVOG0256, NCVOG0262, NCVOG1060, NCVOG1088, NCVOG1115, NCVOG1117, NCVOG1122, NCVOG1127 and NCVOG1192), which are usually present at low copy numbers across all published NCLDV genomes, a duplication ratio was calculated as follows. The total number of copies of the 16 NCVOGs in the respective GVMAG was divided by the total number of unique observations of the 16 NCVOGs. GVMAGs with a duplication ratio higher than three were excluded from the dataset. We then used Diamond BLASTp^47^ against the NCBI non-redundant (nr) database (August 2018) and assigned a taxonomic affiliation on the basis of best BLASTp hits against Archaea, Bacteria, Eukaryota, phages or other viruses (including NCLDVs) to proteins using an *E*-value cut-off of 1 × 10^−5^. Best hits of query proteins to proteins derived from MAGs from the *Tara* Mediterranean metagenome binning survey^57^ were disregarded owing to the high number of misclassified genomes in this dataset. Proteins without a hit in the NCBI nr database were labelled as ‘Unknown’. We then applied filters to remove contigs from GVMAGs on the basis of the distribution of taxonomic affiliation of best blast hits (Supplementary Table 7). Finally, alignments were built with mafft^48^ (v.7.294b) for NCVOG0023, NCVOG0038, NCVOG0076, NCVOG0249 and NCVOG0262. Positions with 90% or more gaps were removed from the alignments with trimal^58^ (v.1.4). Protein alignments were concatenated and a species tree constructed with IQ-tree^59^ (LG + F + R8, v.1.6.10). The phylogenetic tree was then manually inspected and for each clade outliers were removed on the basis of the presence, absence and copy numbers of 20 conserved NCVOGs^16^, duplication factor (see above), coding density, GC content and genome size. In addition, GVMAGs that represented singletons on long branches were manually removed. The filtered dataset was then clustered together with all available NCLDV reference genomes (December 2018) using average nucleotide identities of greater than 95% and an alignment fraction of at least 50% with FastANI^60^ (v.1.1). For each 95% average nucleotide identity cluster the 6 NCVOGs^16^ with the on-average longest amino acid sequences (NCVOG0022, NCVOG0023, NCVOG0038, NCVOG0059, NCVOG0256 and NCVOG1117) were subjected to a within-cluster all-versus-all BLASTp. GVMAGs that had any full-length 100% identity hits between any of these maker proteins to other cluster members were removed from the dataset as potential duplicates. Duplicate GVMAGs originating from the conventional binning approach were removed first and GVMAGs with the largest assembly size were retained.

### GVMAG quality on the basis of estimated completeness and contamination

Estimation of the quality of MAGs is critical for their interpretation and use in downstream applications. Standards exist for bacterial and archaeal MAGs that have proposed a three-tier classification (high, medium or low quality) based on estimated genome completeness and contamination^55^. These completeness and contamination metrics are typically calculated on the basis of a set of universal single-copy marker genes. A set of conserved genes in the NCLDV are the NCVOGs^16^, of which a subset has been shown to be probably vertically inherited^16^ (NCVOG20, Supplementary Table 2). We calculated for each superclade the average number of NCVOG20 present either as a single copy or as multiple copies (Supplementary Table 3). We then compared the number of observed single- and multicopy NCVOG20 in every GVMAG to the mean number of observations in the respective superclade. Considering the high genome plasticity of NCLDVs^2,61^, we tolerated a deviation from the mean by a factor of 1.2, which was considered low contamination, and a factor of 2 was considered medium contamination (Extended Data Fig. 4 and Supplementary Table 4). Higher deviations from the superclade mean were potentially caused by a non-clonal composition of the GVMAG; these were, as a consequence, considered to be of high contamination. We also estimated completeness on the basis of the presence of the NCVOG20 compared with other members of the respective superclade. The presence of 90% or more of the NCVOG20 compared with the superclade mean resulted in a classification as high quality in terms of completeness. If at least 50% of NCVOG20 were present in a GVMAG then the respective GVMAG was classified as medium quality in terms of estimated completeness, or low if less than 50% of NCVOG20 were present (Extended Data Fig. 4 and Supplementary Table 4). The final GVMAG quality was determined on the basis of a combination of contamination and completeness (Supplementary Table 8). Additional criteria to assign GVMAGs to the high-quality category were the presence of no more than 30 contigs, a minimum assembly size of 100 kb and the presence of at least one contig with a length greater than 30 kb. To assign a GVMAG to the medium-quality category were the presence no more than 50 contigs, a minimum assembly size of 100 kb and the presence of at least one contig with a length greater than 15 kb.

### Annotation of GVMAGs

Gene calling was performed with GeneMarkS using the virus model^62^. For functional annotation proteins were subject to BLASTp against previously established NCVOGs^16^ and the NCBI nr database (May 2019) using Diamond (v.0.9.21) BLASTp^47^ with an *E*-value cut-off of 1.0 × 10^−5^. In addition, protein domains were identified by pfam_scan.pl (v.1.6) against Pfam-A^63^ (v.29.0), and rRNAs and introns were identified with cmsearch using the Infernal package^64^ (v.1.1.1) against the Rfam database^65^ (v.13.0). No rRNA genes were detected in the final set of GVMAGs. The eggNOG mapper^66^ (v.1.0.3) was used to assign functional categories to NCLDV proteins. Protein families were inferred with PorthoMCL^67^ (version of December 2018) with default settings.

### Survey of the NCLDV MCP

We used hmmsearch (v.3.1b2, http://hmmer.org/) optimized for the supercomputer Cori^44^ to identify all copies of MCP encoded in the final set of GVMAGs and NCLDV reference genomes. Proteins were extracted and multiple sequence alignments were created with mafft^48^ (v.7.294b) for 74 NCLDV lineages with at least 5 copies of MCP. For each lineage-specific MCP alignment, we inferred models with hmmbuild (v.3.1b2, http://hmmer.org/). Using these models, the modified version of hmmsearch (v.3.1b2, http://hmmer.org/)^44^ was used to identify all MCPs in the entire set of metagenomes (IMG/M^43^, June 2018), MCPs with identical amino acid sequences were excluded as potential duplicates. A logistic-regression-based classifier (sklearn LogisticRegression, solver = ‘lbfgs’, multi_class = ‘ovr’) was trained for each NCLDV lineage taking into account the score distribution of all lineage MCPs hits against the entire set of lineage-specific MCP models. The accuracy of the classifier was 0.861. Unbinned metagenomic MCPs were assigned to NCLDV lineages if the classifier returned a probability greater than 50% (sklearn predict_proba), or as ‘novel’ if the probability was 50% or below. We then normalized the environmental MCP counts on the basis of the observed average copy number of MCP in GVMAGs and reference genomes in the respective lineage. Distribution of NCLDV lineages on the basis of MCPs was projected on a world map with Python 3/basemap on the basis of coordinates provided in IMG metagenomes^43^.

### NCLDV species tree

To build a species tree of the extended NCLDV, viral genomes with at least three out of five core NCVOGs^16^ were selected: DNA polymerase elongation subunit family B (NCVOG0038), D5-like helicase-primase (NCVOG0023), packaging ATPase (NCVOG0249), DNA or RNA helicases of superfamily II (NCVOG0076), and poxvirus late transcription factor VLTF3-like (NCVOG0262). The NCVOGs were identified with hmmsearch (version 3.1b2, http://hmmer.org/) using an *E*-value cut-off of 1 × 10^−10^, extracted and aligned using mafft^48^ (v.7.294b). Columns with less than 10% sequence information were removed from the alignment with trimal^58^. The species tree was then calculated on the basis of the concatenated alignment of all five proteins with IQ-tree^59^ (v.1.6.10) with ultrafast bootstrap^68^ and LG + F + R8 as suggested by model test as the best-fit substitution model^69^. The percentage increase in phylogenetic diversity^70^ was calculated on the basis of the difference of the sum of branch lengths of the phylogenetic species trees of the NCLDV including the GVMAGs compared with a NCLDV species tree calculated from published NCLDV reference genomes (*n* = 205, no dereplication based on the average nucleotide identity) with IQ-tree as described above. Phylogenetic trees were visualized with iTol^71^ (v.5). Genus or subfamily level lineages were defined on the basis of their monophyly in the species tree and presence or absence pattern of conserved NCVOGs (Supplementary Table 4). If no viral isolates were present in the respective monophyletic clade we designated it MGVL. Neighbouring lineages with isolates and MGVLs were further combined under the working term superclade. Branch lengths separating clades differ based on the density of sampled viruses.

### Protein trees

Target proteins were extracted from NCLDV genomes and used to query the NCBI nr database (June 2018) with Diamond BLASTp^47^. The top-50 hits per query were extracted, merged with queries, dereplicated on the basis of protein accession number and aligned with MAFFT (-linsi, v.7.294b)^48^, trimmed with trimal^58^ (removal of positions with more than 90% of gaps) and maximum-likelihood phylogenetic trees inferred with IQ-tree^59^ (multicore v.1.6.10) using ultrafast bootstrap^68^ and the model suggested by the model test feature implemented in IQ-tree^69^ based on Bayesian information criterion. Selected models are indicated in the legend of Extended Data Fig. 8. Owing to its size, the phylogenetic tree for ABC transporter was inferred with FastTree^72^ (v.2.1.10) LG and can be accessed at https://bitbucket.org/berkeleylab/mtg-gv-exp/. Phylogenetic trees were visualized with iTol^71^ (v.5). Information on functional genes including parent contigs is provided in Supplementary Table 5.

### Virus–host linkage through HGT

To generate a cellular nr database, all non-cellular sequences and sequences from the *Tara* Mediterranean genome study^57^ were removed from the NCBI nr database. All proteins in the NCLDV genomes were then subjected to Diamond BLASTp^47^ against the cellular nr database using an *E*-value cut-off of 1 × 10^−50^, an alignment fraction of 50% and a minimum sequence identity of 50%. Best blast hits within the same lineage were removed. Proteins that had a hit in cellular nr with a lower *E* value compared with hits in the NCLDV blast database were considered HGT candidates. The total number of best hits from lineage pan-proteomes against defined groups of Eukaryotes were then used as edge weights to build an HGT network. The network was created in Gephi (v.0.92)^73^ using a force layout and filtered at an edge weight of 2. Pfam annotations of HGT candidates were based on the most commonly detected domains and functional categories were assigned with the eggNOG Mapper (v.1.03)^66^. Information on HGT candidates including parent contigs is provided in Supplementary Table 6. The number of HGT linkages was limited by the available of reference genomes and the stringency applied.

### Reporting summary

Further information on research design is available in the Nature Research Reporting Summary linked to this paper.

## Online content

Any methods, additional references, Nature Research reporting summaries, source data, extended data, supplementary information, acknowledgements, peer review information; details of author contributions and competing interests; and statements of data and code availability are available at 10.1038/s41586-020-1957-x.

## Supplementary information

Supplementary Information Contains supplementary texts 1 and 2 that provide additional information on the NCDLV classifier and the presence of genes with putative roles in photosynthesis, and supplementary references.

Reporting Summary

Supplementary Data 1 | Maximum likelihood phylogeny and genome features of superclades SC1-SC10 This file contains a collection of pdfs of phylogenetic trees for the different superclades. Branches in red indicate Nucleocytoplasmic Large DNA Virus (NCLDV) genomes derived from isolates. Lineage affiliation is indicated in shades of grey. Tracks from the inside to the outside show assembly size in bp, GC in %, coding density in %, number of contigs, environmental origin and copy numbers of conserved Nucleocytoplasmic Virus Orthologous Genes (NCVOGs). Yellow filled circles indicate branch support of > 90 (IQ-tree ultrafast bootstrap). The phylogenetic trees are also provided under the project “GVMAGs” at https://itol.embl.de/shared/fmschulz.

Supplementary Table 1 | Genome features of Giant Virus Metagenome Assembled Genomes (GVMAGs) The table provides information on GVMAG quality, environmental origin and accession numbers of underlying data in SRA, NCBI BioSample, NCBI BioProject, NCBI Genbank and IMG/ JGI Genome Portal (https://genome.jgi.doe.gov/portal).

Supplementary Table 2 | Annotation of Nucleocytoplasmic Virus Orthologous Genes (NCVOGs).

Supplementary Table 3 | Copy numbers of 20 conserved Nucleocytoplasmic Virus Orthologous Genes (NCVOGs) in Giant Virus Metagenome Assembled Genomes (GVMAGs).

Supplementary Table 4 | Estimated quality of Giant Virus Metagenome Assembled Genomes (GVMAGs) Completeness and contamination estimates based on copy numbers 20 conserved Nucleocytoplasmic Virus Orthologous Genes (NCVOGs) compared to the superclade average.

Supplementary Table 5 | Contig composition for selected functional genes Distribution of best blastp hits against NCBI non-redundant for genes on contigs encoding for functional genes which are part of the analysis underlying Fig. 2. In addition, Pfam-A annotations of respective proteins are provided.

Supplementary Table 6 | Contig composition for eukaryotic HGT candidates Distribution of best blastp hits against NCBI non-redundant for genes on contigs encoding for eukaryotic HGT candidates which are part of the analysis underlying Fig. 3, and bacterial or archaeal HGT candidates. In addition, Pfam-A annotations of respective proteins are provided.

Supplementary Table 7 | Filters applied to remove potential contaminant contigs from GVMAGs. For each contig filtering was performed based on taxonomic distribution of best blastp hits in the NCBI nr database. Contigs with values greater than the indicated values highlighted in grey and at the same time smaller than the indicated values highlighted in white were removed from the dataset.

Supplementary Table 8 | Criteria used to define the quality of GVMAGs

## Data Availability

All GVMAGs of estimated high and medium quality with an N50 of greater than 50 kb and estimated low contamination have been deposited at NCBI GenBank as MN738741–MN741037 under BioProject ID PRJNA588800. Nucleotide and protein sequences of GVMAGs can be directly downloaded from https://genome.jgi.doe.gov/portal/GVMAGs and https://figshare.com/s/14788165283d65466732, and will be available in the Integrated Microbial Genome/Virus (IMG/VR) system^74^ at time of the v.3.0 release. All of the sequence data and metadata from the samples used in this study can further be accessed through the IMG/M system^43^ (https://img.jgi.doe.gov) and NCBI SRA using the metagenome identifiers provided in Supplementary Table 1. Sequence alignments, phylogenetic trees and other data underlying this study can be downloaded from https://genome.jgi.doe.gov/portal/GVMAGs.

## Code availability

The NCLDV classifier can be obtained from https://bitbucket.org/berkeleylab/mtg-gv-exp/.
