# Supplementary material for: Giant virus diversity and host interactions through global metagenomics
Source: Nature. 2020 Jan 22;578(7795):432–6. doi: 10.1038/s41586-020-1957-x (PMC7162819; doi:10.1038/s41586-020-1957-x)
Supplement: Supplementary file 1 — Contains supplementary texts 1 and 2 that provide additional information on the NCDLV classifier and the presence of genes with putative roles in photosynthesis, and supplementary references. [file 41586_2020_1957_MOESM1_ESM.pdf]

nature

https://doi.org/10.1038/s41586-020-1957-x

Supplementary information

# Giant virus diversity and host interactions through global metagenomics

In the format provided by the authors and unedited

Frederik Schulz✉, Simon Roux, David Paez-Espino, Sean Jungbluth, David A. Walsh, Vincent J. Denef, Katherine D. McMahon, Konstantinos T. Konstantinidis, Emiley A. Eloe-Fadrosh, Nikos C. Kyrpides & Tanja Woyke✉

**Supplementary Text 1**

**De novo RBS prediction can be leveraged to distinguish NCLDV contigs from other microbial and viral genomes**

A comparison of gene predictions on known NCLDV genomes and reference genomes against eukaryotic, archaeal, bacterial, and non-NCLDV viral genomes revealed specific features of NCLDV that could be used in addition to similarity-based methods for identifying NCLDV sequences in a mixed metagenome. Specifically, when using a gene predictor designed for prokaryotic genomes[49], the density of predicted genes was significantly higher for bacterial, archaeal, and NCLDV genomes, as compared to eukaryotic genomes (Extended Data Figure 2, k-s test p-value < 2.2e-16, effect size > 3). The only exceptions were pandoraviruses, for which the predicted gene density was significantly lower (k-s test p-value = 1.5e-97, effect size = 3.1) and comparable to the gene density of non-NCLDV eukaryotic virus genomes.

In addition to the gene density, we also explored whether the ribosomal binding sites (RBS) motifs predicted by Prodigal could further separate bacterial and archaeal genomes from NCLDV genomes. We reasoned that, since NCLDV rely on eukaryotic ribosomes for translation, the motifs detected on these genomes might be different from the motifs typically associated with bacterial and archaeal ribosomes. Importantly, the predicted motifs may not represent true binding sites for ribosomes but instead other conserved regions (e.g. transcription-related motifs) found upstream of predicted start codons in these different genomes. By calculating the average frequency of different types of motifs (see Methods), we observed that NCLDV genomes were enriched in predicted motifs including 'TA' repeats, while most bacterial and archaeal genomes are enriched in canonical RBS motifs, i.e. variations of the AGGAGG Shine-Dalgarno (SD) sequence (Extended Data Figure 2). Consistent with the low number of genes predicted, Prodigal tended to not predict any RBS motif in eukaryotic genomes, including putative NCLDV hosts such as protists and fungi. Several groups of bacteria and archaea including extremophiles microbes and members of the Bacteroidetes phylum are known to harbor unusual RBS motifs [75] and were accordingly associated with non-SD motifs, however none of these groups displayed the NCLDV 'TA' motif as most abundant (Extended Data Figure 2). Finally, among NCLDV, Pandoraviruses were not associated with the same 'TA' motif but instead displayed a mix of 'no RBS' and 'OnlyA' motifs. Combined with their lower gene density, this suggests that pandoravirus genomes harbor unique features compared to other NCLDV.

Based on these specific features identified for gene prediction on NCLDV genomes, we built random forest classifiers to identify contigs originating from NCLDV in metagenome assemblies or genome bins. For complete genomes, a random forest classifier based on features and motif predicted de novo by Prodigal was able to correctly discriminate NCLDV contigs from any other type of genome tested here (Extended Data Figure 2). In 10-fold cross validations, 79% of NCLDV genomes were classified as 'NCLDV origin' with a score ≥ 0.2, while < 2% of genomes

displayed a score ≥ 0.2 in any other group (Extended Data Figure 2). A separate classifier was built for metagenomic contigs and trained on short (10-20 kb) fragments of the reference genomes (see Methods). Although less efficient than for the complete genome, the automatic classifier for metagenome contigs was still able to distinguish NCLDV from any other type of sequences (Extended Data Figure 2). Concretely, > 60% of NCLDV contigs were classified with a score ≥ 0.3, while ≤ 10% of contigs displayed a score ≥ 0.3 in any other group. The microbial genomes with unusual RBS motifs (see above) were the principal source of false-positive detections (i.e. non-NCLDV sequence with a high score NCLDV origin prediction, Extended Data Figure 2). Hence these will represent the main sources of errors when extracting NCLDV contigs from metagenome assemblies using this classifier.

**Supplementary Text 2**

**Giant virus-encoded genes with potential roles in chlorophyll-based photosynthesis**

In total 11% of GVMAGs encoded for genes with potential roles in chlorophyll-based photosynthetic processes. Most commonly found photosynthesis genes in the GVMAGs were homologs of eukaryotic light harvesting proteins which are predicted to bind to the pigment chlorophyll A or B[76]. These genes were present in 222 GVMAGs, predominantly in members of superclades 9 and 10, which are both part of the extended Mimiviruses. Among known members of both superclades are algae-infecting viruses, such as *Tetraselmis* virus, *Aureococcus anaphaegensis* virus and *Chrysomulina ericina* virus but also viruses which infect heterotrophic protists such as choanoflagellates[14]. In our phylogenetic analysis the majority of NCLDV chlorophyll AB binding proteins group together in a well separated monophyletic clade with the closest related cellular light harvesting protein found in the alveolate *Vitrella brassicaformis* (Extended Data Figure 8). Importantly, there are nine additional clades of NCLDV chloroAB genes which branch closer to their cellular counterparts and were likely derived by more recent horizontal gene transfer from diverse algal hosts, such as *Thalassiosira*, *Bigelowiella* and *Aureococcus* (Extended Data Fig. 8). Other photosynthesis genes were less commonly found and more divergent from their cellular homologs; 7 GVMAGs encoded for putative chlorophyllases, membrane proteins which play important roles in the breakdown of chlorophyll to chlorophyllides [77] and 4 contained putative bestrophins, which potentially function as ion channels in the thylakoid membrane of photosynthetic cells[78]. The role of photosynthetic genes in NCLDVs might be comparable to similar genes in cyanophages[79]. These phages which infect Cyanobacteria typically complement their host's physiology by expressing virus-encoded genes encoding PSII core reaction-center proteins, in order to sustain active photosynthesis and energy generation in the cell even after the shut down of host genes expression due to viral infection[80,81]. Beyond maintaining the function of host cell photosynthetic systems, the NCLDV chlorophyll AB binding proteins could potentially also modulate the accessible light spectrum of the host. As these are just speculations, the ultimate role of NCLDV-encoded photosynthesis genes in host interaction remains yet to be elucidated.

**References**

68. Hoang, D. T., Chernomor, O., von Haeseler, A., Minh, B. Q. & Vinh, L. S. UFBoot2: Improving the Ultrafast Bootstrap Approximation. *Mol. Biol. Evol.* **35**, 518–522 (2018).

69. Kalyaanamoorthy, S., Minh, B. Q., Wong, T. K. F., von Haeseler, A. & Jermiin, L. S. ModelFinder: fast model selection for accurate phylogenetic estimates. *Nat. Methods* **14**, 587–589 (2017).

70. Wu, D. *et al.* A phylogeny-driven genomic encyclopaedia of Bacteria and Archaea. *Nature* **462**, 1056–1060 (2009).

71. Letunic, I. & Bork, P. Interactive tree of life (iTOL) v3: an online tool for the display and annotation of phylogenetic and other trees. *Nucleic Acids Res.* **44**, W242–5 (2016).

72. Price, M. N., Dehal, P. S. & Arkin, A. P. FastTree 2--approximately maximum-likelihood trees for large alignments. *PLoS One* **5**, e9490 (2010).

73. Bastian, M., Heymann, S., Jacomy, M. & Others. Gephi: an open source software for exploring and manipulating networks. *Icwsm* **8**, 361–362 (2009).

74. Paez-Espino, D. *et al.* IMG/VR v.2.0: an integrated data management and analysis system for cultivated and environmental viral genomes. *Nucleic Acids Res.* **47**, D678–D686 (2019).

75. Wegmann, U., Horn, N. & Carding, S. R. Defining the bacteroides ribosomal binding site. *Appl. Environ. Microbiol.* **79**, 1980–1989 (2013).

76. Green, B. R., Pichersky, E. & Kloppstech, K. Chlorophyll a/b-binding proteins: an extended family. *Trends Biochem. Sci.* **16**, 181–186 (1991).

77. Tsuchiya, T. *et al.* Cloning of chlorophyllase, the key enzyme in chlorophyll degradation: finding of a lipase motif and the induction by methyl jasmonate. *Proc. Natl. Acad. Sci. U. S. A.* **96**, 15362–15367 (1999).

78. Duan, Z. *et al.* A bestrophin-like protein modulates the proton motive force across the thylakoid membrane inArabidopsis. *Journal of Integrative Plant Biology* **58**, 848–858 (2016).

79. Suttle, C. A. Cyanophages and their role in the ecology of cyanobacteria. in *The ecology of cyanobacteria* 563–589 (Springer, 2000).

80. Sullivan, M. B. *et al.* Prevalence and evolution of core photosystem II genes in marine cyanobacterial viruses and their hosts. *PLoS Biol.* **4**, e234 (2006).

81. Clokie, M. R. J. & Mann, N. H. Marine cyanophages and light. *Environ. Microbiol.* **8**, 2074–2082 (2006).

82. Beck, D. A. C. *et al.* A metagenomic insight into freshwater methane-utilizing communities and evidence for cooperation between the Methylococcaceae and the Methylophilaceae. *PeerJ* **1**, e23 (2013).

83. Espínola, F. *et al.* Metagenomic Analysis of Subtidal Sediments from Polar and Subpolar Coastal Environments Highlights the Relevance of Anaerobic Hydrocarbon Degradation Processes. *Microb. Ecol.* **75**, 123–139 (2018).

84. Wilhelm, R. C., Hanson, B. T., Chandra, S. & Madsen, E. Community dynamics and functional characteristics of naphthalene-degrading populations in contaminated surface sediments and hypoxic/anoxic groundwater. *Environ. Microbiol.* **20**, 3543–3559 (2018).

85. Teeling, H. *et al.* Recurring patterns in bacterioplankton dynamics during coastal spring algae blooms. *Elife* **5**, e11888 (2016).

86. Garcia, S. L. *et al.* Model Communities Hint at Promiscuous Metabolic Linkages between Ubiquitous Free-Living Freshwater Bacteria. *mSphere* **3**, (2018).

87. Bendall, M. L. *et al.* Genome-wide selective sweeps and gene-specific sweeps in natural bacterial populations. *ISME J.* **10**, 1589–1601 (2016).

88. Denef, V. J., Mueller, R. S., Chiang, E., Liebig, J. R. & Vanderploeg, H. A. Chloroflexi CL500-11 Populations That Predominate Deep-Lake Hypolimnion Bacterioplankton Rely on Nitrogen-Rich Dissolved Organic Matter Metabolism and C1 Compound Oxidation. *Appl. Environ. Microbiol.* **82**, 1423–1432 (2015).

89. Hawley, A. K. *et al.* A compendium of multi-omic sequence information from the Saanich Inlet water column. *Sci Data* **4**, 170160 (2017).

90. Reddy, A. P. *et al.* Discovery of microorganisms and enzymes involved in high-solids decomposition of rice straw using metagenomic analyses. *PLoS One* **8**, e77985 (2013).

91. Ganesh, S. *et al.* Single cell genomic and transcriptomic evidence for the use of alternative nitrogen substrates by anammox bacteria. *ISME J.* **12**, 2706–2722 (2018).

92. Tsementzi, D. *et al.* SAR11 bacteria linked to ocean anoxia and nitrogen loss. *Nature* **536**, 179–183 (2016).

93. Sorensen, J. W., Dunivin, T. K., Tobin, T. C. & Shade, A. Ecological selection for small microbial genomes along a temperate-to-thermal soil gradient. *Nat Microbiol* **4**, 55–61 (2019).

94. Graham, E. B. *et al.* Multi'omics comparison reveals metabolome biochemistry, not microbiome composition or gene expression, corresponds to elevated biogeochemical function in the hyporheic zone. *Sci. Total Environ.* **642**, 742–753 (2018).

95. Maresca, J. A., Miller, K. J., Keffer, J. L., Sabanayagam, C. R. & Campbell, B. J. Distribution and Diversity of Rhodopsin-Producing Microbes in the Chesapeake Bay. *Appl.*

*Environ. Microbiol.* **84**, (2018).

96. Shiller, A. M., Chan, E. W., Joung, D. J., Redmond, M. C. & Kessler, J. D. Light rare earth element depletion during Deepwater Horizon blowout methanotrophy. *Scientific Reports* **7**, (2017).

97. Dalcin Martins, P. *et al.* Viral and metabolic controls on high rates of microbial sulfur and carbon cycling in wetland ecosystems. *Microbiome* **6**, 138 (2018).

98. Tran, P. *et al.* Microbial life under ice: Metagenome diversity and in situ activity of Verrucomicrobia in seasonally ice-covered Lakes. *Environ. Microbiol.* **20**, 2568–2584 (2018).

99. Colatriano, D. *et al.* Genomic evidence for the degradation of terrestrial organic matter by pelagic Arctic Ocean Chloroflexi bacteria. *Commun Biol* **1**, 90 (2018).

100. Daly, R. A. *et al.* Viruses control dominant bacteria colonizing the terrestrial deep biosphere after hydraulic fracturing. *Nat Microbiol* **4**, 352–361 (2019).

101. Roux, S. *et al.* Ecogenomics and potential biogeochemical impacts of globally abundant ocean viruses. *Nature* **537**, 689–693 (2016).

102. Grettenberger, C. L. *et al.* Insights into the evolution of oxygenic photosynthesis from a phylogenetically novel, low-light cyanobacterium. *bioRxiv* 334458 (2018). doi:10.1101/334458

103. Nowinski, B. *et al.* Microbial metagenomes and metatranscriptomes during a coastal phytoplankton bloom. *Sci Data* **6**, 129 (2019).

104. Wu, Y.-W. *et al.* Ionic Liquids Impact the Bioenergy Feedstock-Degrading Microbiome and Transcription of Enzymes Relevant to Polysaccharide Hydrolysis. *mSystems* **1**, (2016).

105. Krüger, K. *et al.* In marine Bacteroidetes the bulk of glycan degradation during algae blooms is mediated by few clades using a restricted set of genes. *ISME J.* **13**, 2800–2816 (2019).

106. D'haeseleer, P. *et al.* Metagenomic analysis of intertidal hypersaline microbial mats from Elkhorn Slough, California, grown with and without molybdate. *Stand. Genomic Sci.* **12**, 67 (2017).

107. Lee, L. L. *et al.* Genus-Wide Assessment of Lignocellulose Utilization in the Extremely Thermophilic Genus Caldicellulosiruptor by Genomic, Pangenomic, and Metagenomic Analyses. *Appl. Environ. Microbiol.* **84**, (2018).

108. Anantharaman, K. *et al.* Expanded diversity of microbial groups that shape the dissimilatory sulfur cycle. *ISME J.* **12**, 1715–1728 (2018).

109. Swan, B. K. *et al.* Potential for chemolithoautotrophy among ubiquitous bacteria lineages in the dark ocean. *Science* **333**, 1296–1300 (2011).

110. Vavourakis, C. D. *et al.* Metagenomic Insights into the Uncultured Diversity and Physiology of Microbes in Four Hypersaline Soda Lake Brines. *Front. Microbiol.* **7**, 211 (2016).

111. Vavourakis, C. D. *et al.* A metagenomics roadmap to the uncultured genome diversity in hypersaline soda lake sediments. *Microbiome* **6**, 168 (2018).

112. Pesant, S. *et al.* Open science resources for the discovery and analysis of Tara Oceans data. *Sci Data* **2**, 150023 (2015).
