# Supplementary material for: Giant virus diversity and host interactions through global metagenomics
Source: Nature. 2020 Jan 22;578(7795):432–6. doi: 10.1038/s41586-020-1957-x (PMC7162819; doi:10.1038/s41586-020-1957-x)
Supplement: Supplementary file 2 — Reporting Summary [file 41586_2020_1957_MOESM2_ESM.pdf]

nature research

Corresponding author(s): Frederik Schulz
Tanja Woyke

Last updated by author(s): Nov 23, 2019

Reporting Summary

Nature Research wishes to improve the reproducibility of the work that we publish. This form provides structure for consistency and transparency in reporting. For further information on Nature Research policies, see Authors & Referees and the Editorial Policy Checklist .

Statistics

For all statistical analyses, confirm that the following items are present in the figure legend, table legend, main text, or Methods section.

n/a	Confirmed	
☒	☐	The exact sample size (*n*) for each experimental group/condition, given as a discrete number and unit of measurement
☒	☐	A statement on whether measurements were taken from distinct samples or whether the same sample was measured repeatedly
☒	☐	The statistical test(s) used AND whether they are one- or two-sided<br>*Only common tests should be described solely by name; describe more complex techniques in the Methods section.*
☒	☐	A description of all covariates tested
☒	☐	A description of any assumptions or corrections, such as tests of normality and adjustment for multiple comparisons
☒	☐	A full description of the statistical parameters including central tendency (e.g. means) or other basic estimates (e.g. regression coefficient) AND variation (e.g. standard deviation) or associated estimates of uncertainty (e.g. confidence intervals)
☒	☐	For null hypothesis testing, the test statistic (e.g. *F*, *t*, *r*) with confidence intervals, effect sizes, degrees of freedom and *P* value noted<br>*Give P values as exact values whenever suitable.*
☒	☐	For Bayesian analysis, information on the choice of priors and Markov chain Monte Carlo settings
☒	☐	For hierarchical and complex designs, identification of the appropriate level for tests and full reporting of outcomes
☒	☐	Estimates of effect sizes (e.g. Cohen's *d*, Pearson's *r*), indicating how they were calculated

Our web collection on statistics for biologists contains articles on many of the points above.

Software and code

Policy information about availability of computer code

Data collection	IMG/M June2018<br>NCBI Genbank (June 2018)<br>NCBI non redundant database May 2019<br>Pfam-A (v 29.0)<br>Rfam (v 13.0)
Data analysis	hmmer (version 3.1b2)<br>orthofinder (v2.27)<br>mafft (v7.294b)<br>Prodigal (v2.6.3)<br>MetaBAT (v0.32.4)<br>MetaBAT (v2)<br>CheckM (v1.0.7)<br>trimAL (v1.4)<br>IQ-tree (1.6.10)<br>FastANI (v1.1)<br>Diamond (v0.9.21)<br>Infernal (v1.1.1)<br>pfam_scan.pl (v1.6)<br>iTOL (v5)<br>eggNOG mapper (v1.0.3)<br>FastTree (v.2.1.10)<br>Gephi (v0.92)

Scikit-learn (v0.20.3)
stats package (v4) in R
PorthoMCL (version of December 2018)
NCLDV classifier is available at https://bitbucket.org/berkeleylab/mtg-gv-exp/

For manuscripts utilizing custom algorithms or software that are central to the research but not yet described in published literature, software must be made available to editors/reviewers. We strongly encourage code deposition in a community repository (e.g. GitHub). See the Nature Research guidelines for submitting code & software for further information.

Data

Policy information about availability of data

All manuscripts must include a data availability statement. This statement should provide the following information, where applicable:

- Accession codes, unique identifiers, or web links for publicly available datasets
- A list of figures that have associated raw data
- A description of any restrictions on data availability

All GVMAGs of estimated high and medium quality with an N50 of greater than 50kb and estimated 'low' contamination have been deposited at NCBI Genbank under BioProject ID PRJNA588800. Nucleotide and protein sequences of GVMAGs can be directly downloaded from https://genome.jgi.doe.gov/portal/GVMAGs and will become available in IMG/VR74 at time of the v.3.0 release. All the sequence data and metadata from the samples used in this work can further be accessed through the Integrated Microbial Genomes and Microbiomes (IMG/M) systems43 (https://img.jgi.doe.gov) and NCBI SRA using the metagenome identifiers provided in Supplementary Table 1. Sequence alignments, phylogenetic trees and other data underlying this study can be downloaded from https://genome.jgi.doe.gov/portal/GVMAGs

Field-specific reporting

Please select the one below that is the best fit for your research. If you are not sure, read the appropriate sections before making your selection.

☐ Life sciences ☐ Behavioural & social sciences ☒ Ecological, evolutionary & environmental sciences

For a reference copy of the document with all sections, see nature.com/documents/nr-reporting-summary-flat.pdf

Ecological, evolutionary & environmental sciences study design

All studies must disclose on these points even when the disclosure is negative.

Study description	Recovery of nucleocytoplasmic large DNA virus metagenome assembled genomes from all publicly available metagenome data in IMG (https://img.jgi.doe.gov/)
Research sample	No samples were taken for this study, subject of this study was all publicly available metagenome data in IMG in June2018 encompassing 8,535 datasets (https://img.jgi.doe.gov/)
Sampling strategy	No samples were taken for this study, all existing publicly available metagenome data in the IMG/M database (https://img.jgi.doe.gov/) in June 2018 was used in this study.
Data collection	The data was collected in June 2018 from the IMG/M database (https://img.jgi.doe.gov/) by Frederik Schulz
Timing and spatial scale	Metagenomic data was generated between 2008 and 2018 by the DOE JGI User Community and Tara Oceans
Data exclusions	For unpublished metagenome datasets used in this study, PIs are either included as co-authors, or PIs were asked for permission and if permission was denied the datasets were excluded from the analysis
Reproducibility	This experiment has not been reproduced but can be reproduced with the methods outlined in the manuscript.
Randomization	Randomization in not relevant in this study as all available data has been mined for nucleocytoplasmic large DNA virus metagenome assembled genomes
Blinding	Blinding was not relevant for this study as the same methods have been applied to the entire data set.

Did the study involve field work? ☐ Yes ☒ No

Reporting for specific materials, systems and methods

We require information from authors about some types of materials, experimental systems and methods used in many studies. Here, indicate whether each material, system or method listed is relevant to your study. If you are not sure if a list item applies to your research, read the appropriate section before selecting a response.

Materials & experimental systems

n/a	Involved in the study
☒	☐ Antibodies
☒	☐ Eukaryotic cell lines
☒	☐ Palaeontology
☒	☐ Animals and other organisms
☒	☐ Human research participants
☒	☐ Clinical data

Methods

n/a	Involved in the study
☒	☐ ChIP-seq
☒	☐ Flow cytometry
☒	☐ MRI-based neuroimaging
