# Supplementary material for: Giant virus diversity and host interactions through global metagenomics
Source: Nature. 2020 Jan 22;578(7795):432–6. doi: 10.1038/s41586-020-1957-x (PMC7162819; doi:10.1038/s41586-020-1957-x)
Supplement: Supplementary file 3 — This file contains a collection of pdfs of phylogenetic trees for the different superclades. Branches in red indicate Nucleocytoplasmic Large DNA Virus (NCLDV) genomes derived from isolates. Lineage affiliation is indicated in shades of grey. Tracks from the inside to the outside show assembly size in bp, GC in %, coding density in %, number of contigs, environmental origin and copy numbers of conserved Nucleocytoplasmic Virus Orthologous Genes (NCVOGs). Yellow filled circles indicate branch support of > 90 (IQ-tree ultrafast bootstrap). The phylogenetic trees are also provided under the project “GVMAGs” at https://itol.embl.de/shared/fmschulz. [file 41586_2020_1957_MOESM3_ESM.pdf]

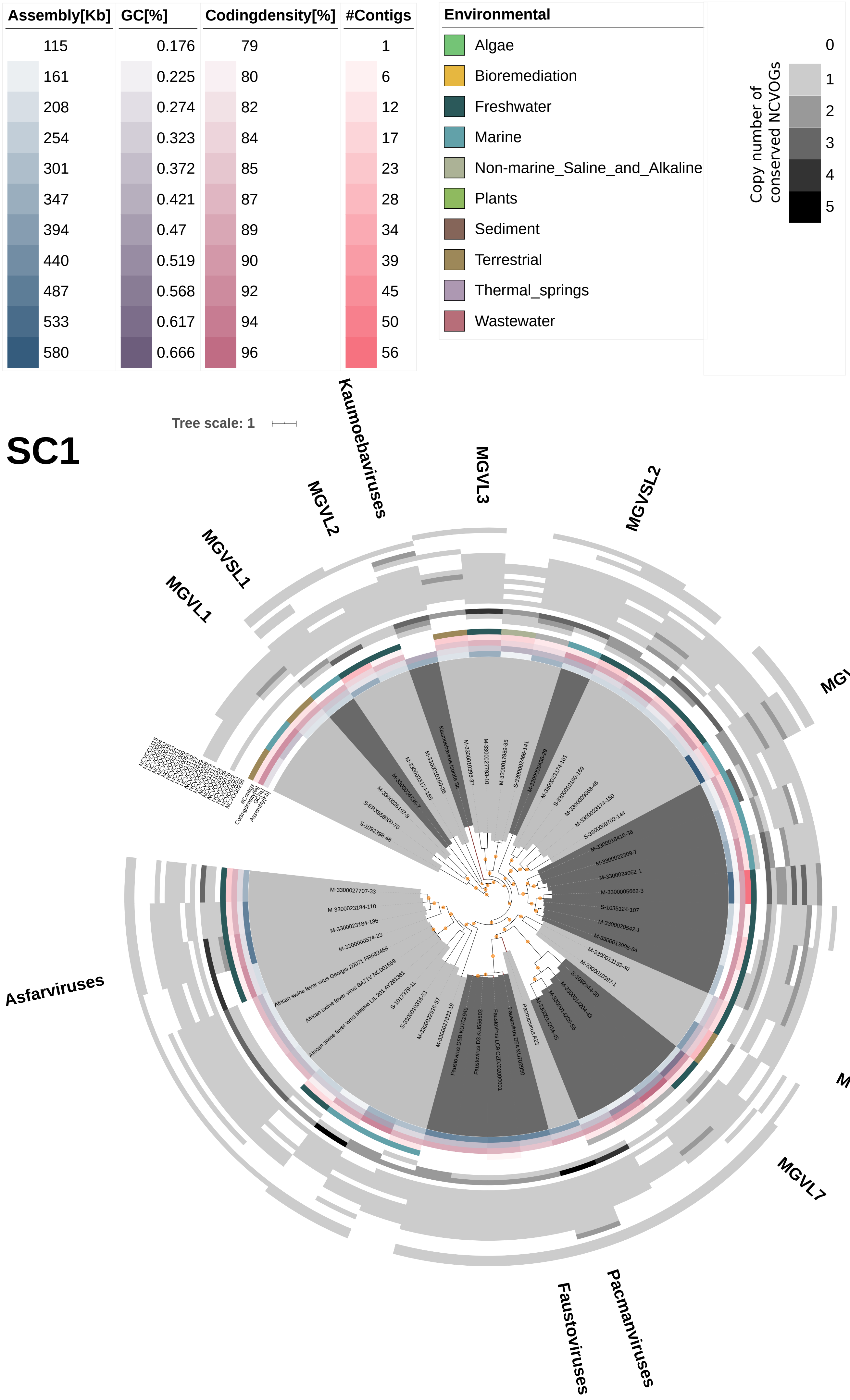

Assembly[Kb]
115
161
208
254
301
347
394
440
487
533
580
GC[%]
0.176
0.225
0.274
0.323
0.372
0.421
0.47
0.519
0.568
0.617
0.666
Codingdensity[%]
79
80
82
84
85
87
89
90
92
94
96
#Contigs
1
6
12
17
23
28
34
39
45
50
56
Environmental
Algae
Bioremediation
Freshwater
Marine
Non-marine_Saline_and_Alkaline
Plants
Sediment
Terrestrial
Thermal_springs
Wastewater
Copy number of conserved NCVOGs
0
1
2
3
4
5
Tree scale: 1
SC1
Kaumoebaviruses
MGVL3
MGVSL2
MGVL2
MGVSL1
MGVL1
MGVL4
MGVL5
MGVL6
MGVL7
Pacmanviruses
Faustoviruses
Asfarviruses
Kaumoebavirus isolate Sc
M-3300010160-26
M-3300023174-165
M-3300024336-7
M-3300026197-8
S-ERX556000-70
S-1092398-48
M-3300010396-37
M-3300027793-10
M-3300017989-35
S-3300002468-141
M-3300009436-29
M-3300023174-161
S-3300010160-169
M-3300009068-46
M-3300023174-150
S-3300009702-144
M-3300018416-36
M-3300022309-7
M-3300024062-1
M-3300005662-3
S-1035124-107
M-3300020542-1
M-3300013005-64
M-3300013133-40
M-3300010397-1
S-1092944-30
M-3300014204-43
M-3300014205-55
M-3300014204-45
Pacmanvirus A23
Faustovirus D5A KU702950
Faustovirus LC9 CZDJ02000001
Faustovirus D3 KU556803
Faustovirus D5B KU702949
M-3300027833-19
M-3300022916-57
S-3300010316-51
S-1017379-11
African swine fever virus Malawi LIL 20/1 AY261361
African swine fever virus BA71V NC001659
African swine fever virus Georgia 2007/1 FR682468
M-3300000574-23
M-3300023184-186
M-3300023184-110
M-3300027707-33
#Contigs
Codingdensity[%]
GC[%]
Assembly[Kb]

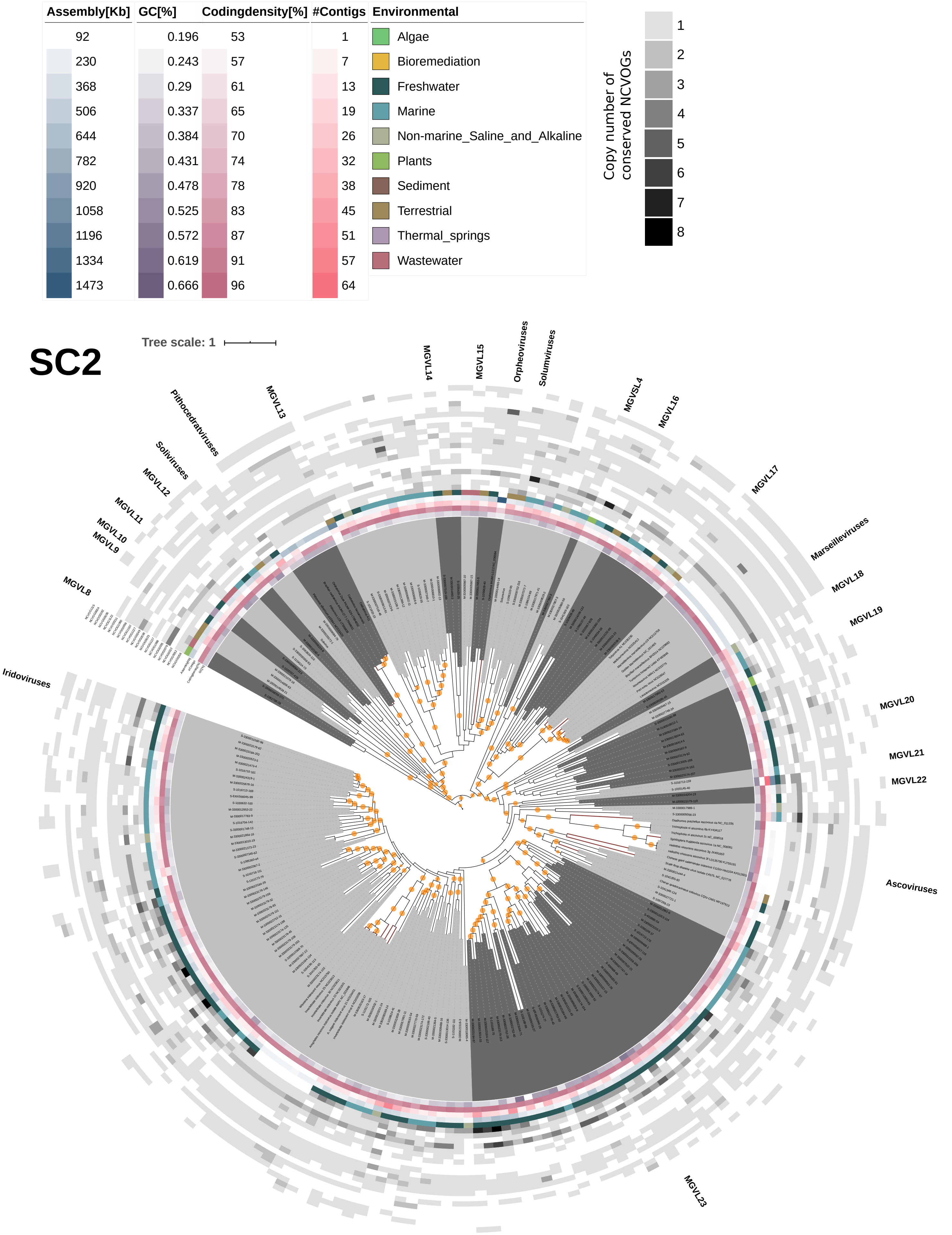
Assembly[Kb]
92
230
368
506
644
782
920
1058
1196
1334
1473
GC[%]
0.196
0.243
0.29
0.337
0.384
0.431
0.478
0.525
0.572
0.619
0.666
Codingdensity[%]
53
57
61
65
70
74
78
83
87
91
96
#Contigs
1
7
13
19
26
32
38
45
51
57
64
Environmental
Algae
Bioremediation
Freshwater
Marine
Non-marine_Saline_and_Alkaline
Plants
Sediment
Terrestrial
Thermal_springs
Wastewater
Copy number of conserved NCVOGs
1
2
3
4
5
6
7
8
SC2
Tree scale: 1
MGVL14
MGVL15
Orpheoviruses
Solumviruses
MGVSL4
MGVL16
MGVL17
Marseilleviruses
MGVL18
MGVL19
MGVL20
MGVL21
MGVL22
Ascoviruses
MGVL23
Iridoviruses
MGVL8
MGVL9
MGVL10
MGVL11
MGVL12
Soliviruses
Pithocedratviruses
MGVL13

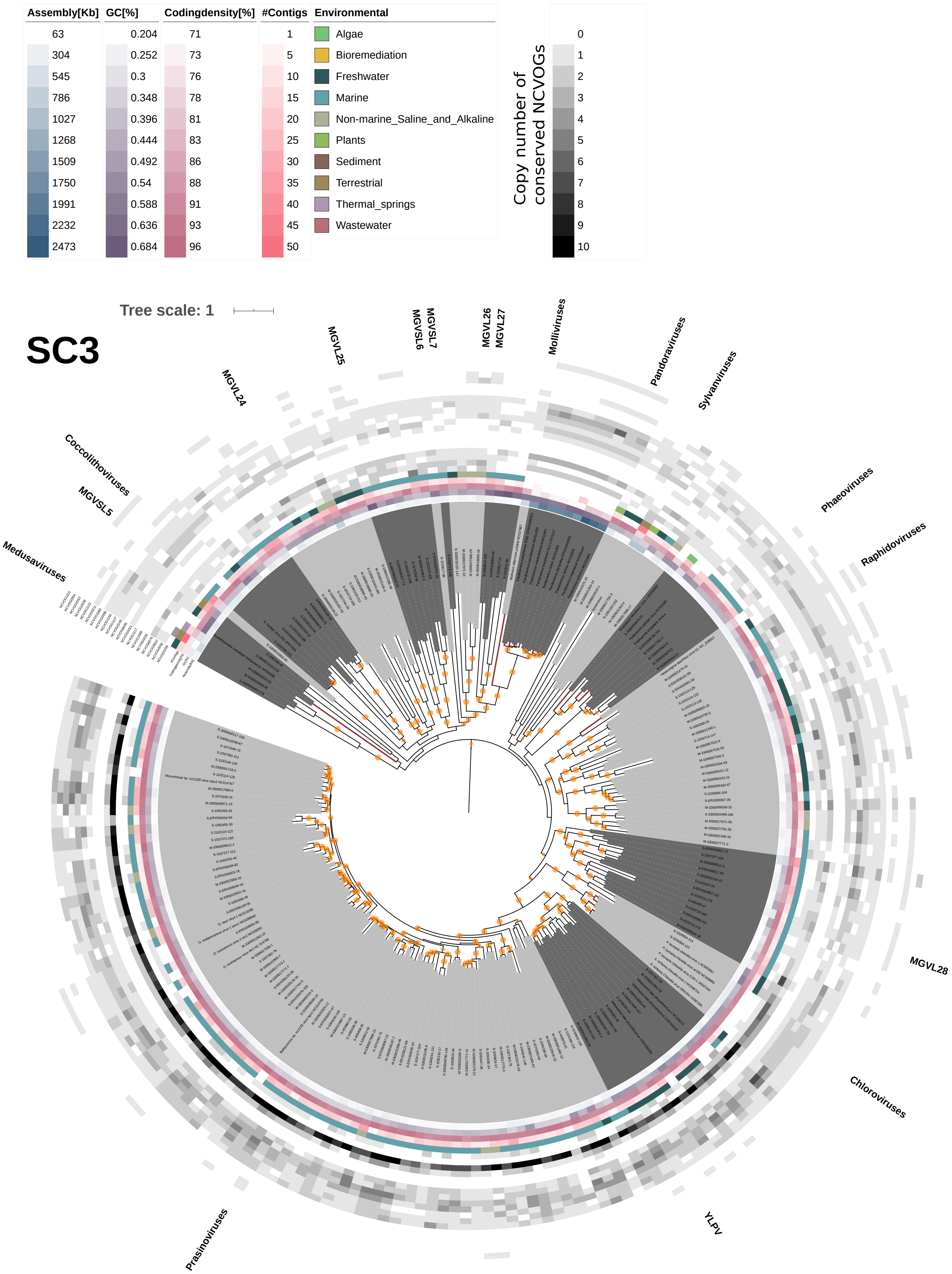

Assembly[Kb]
63
304
545
786
1027
1268
1509
1750
1991
2232
2473
GC[%]
0.204
0.252
0.3
0.348
0.396
0.444
0.492
0.54
0.588
0.636
0.684
Codingdensity[%]
71
73
76
78
81
83
86
88
91
93
96
#Contigs
1
5
10
15
20
25
30
35
40
45
50
Environmental
Algae
Bioremediation
Freshwater
Marine
Non-marine_Saline_and_Alkaline
Plants
Sediment
Terrestrial
Thermal_springs
Wastewater
Copy number of conserved NCVOGs
0
1
2
3
4
5
6
7
8
9
10
Tree scale: 1
SC3
MGVL24
MGVL25
MGVSL6
MGVSL7
MGVL26
MGVL27
Molliviruses
Pandoraviruses
Sylvanviruses
Phaeoviruses
Raphidoviruses
MGVL28
Chloroviruses
YLPV
Prasinoviruses
Medusaviruses
MGVSL5
Coccolithoviruses

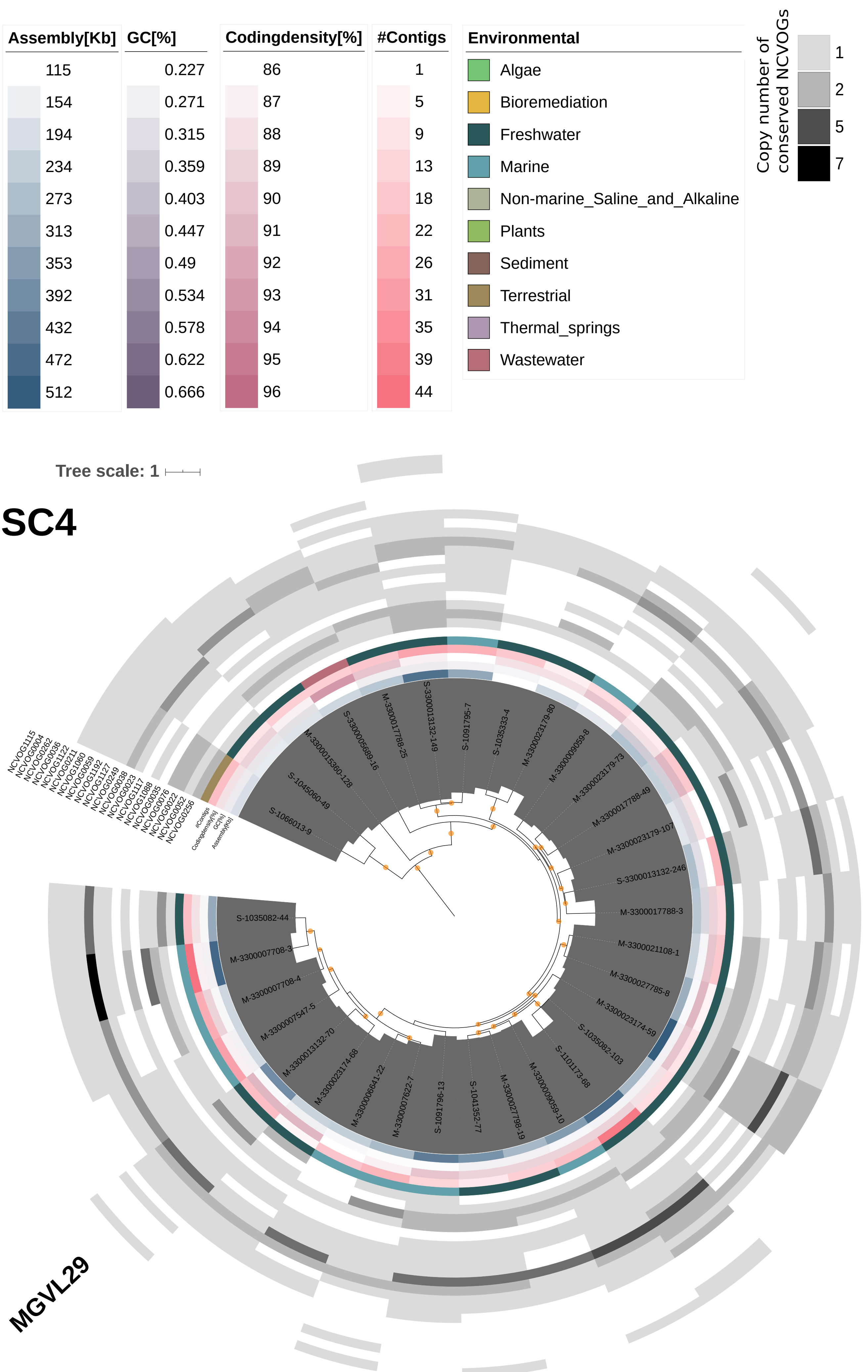
Assembly[Kb]
115
154
194
234
273
313
353
392
432
472
512
GC[%]
0.227
0.271
0.315
0.359
0.403
0.447
0.49
0.534
0.578
0.622
0.666
Codingdensity[%]
86
87
88
89
90
91
92
93
94
95
96
#Contigs
1
5
9
13
18
22
26
31
35
39
44
Environmental
Algae
Bioremediation
Freshwater
Marine
Non-marine_Saline_and_Alkaline
Plants
Sediment
Terrestrial
Thermal_springs
Wastewater
Copy number of conserved NCVOGs
1
2
5
7
Tree scale: 1
SC4
MGVL29
NCVOG1115
NCVOG0004
NCVOG0262
NCVOG0036
NCVOG1122
NCVOG0211
NCVOG1060
NCVOG0059
NCVOG1192
NCVOG1127
NCVOG0249
NCVOG0038
NCVOG0023
NCVOG1117
NCVOG1088
NCVOG0035
NCVOG0076
NCVOG0022
NCVOG0052
NCVOG0256
#Contigs
Codingdensity[%]
GC[%]
Assembly[Kb]
S-1066013-9
S-1045060-49
M-3300015360-128
S-3300005689-16
M-3300017788-25
S-3300013132-149
S-1091795-7
S-1035333-4
M-3300023179-80
M-3300009059-8
M-3300023179-73
M-3300017788-49
M-3300023179-107
S-3300013132-246
M-3300017788-3
M-3300021108-1
M-3300027785-8
M-3300023174-59
S-1035082-103
S-1101173-68
M-3300009059-10
M-3300027798-19
S-1041352-77
S-1091796-13
M-3300007622-7
M-3300006641-22
M-3300023174-68
M-3300013132-70
M-3300007547-5
M-3300007708-4
M-3300007708-3
S-1035082-44

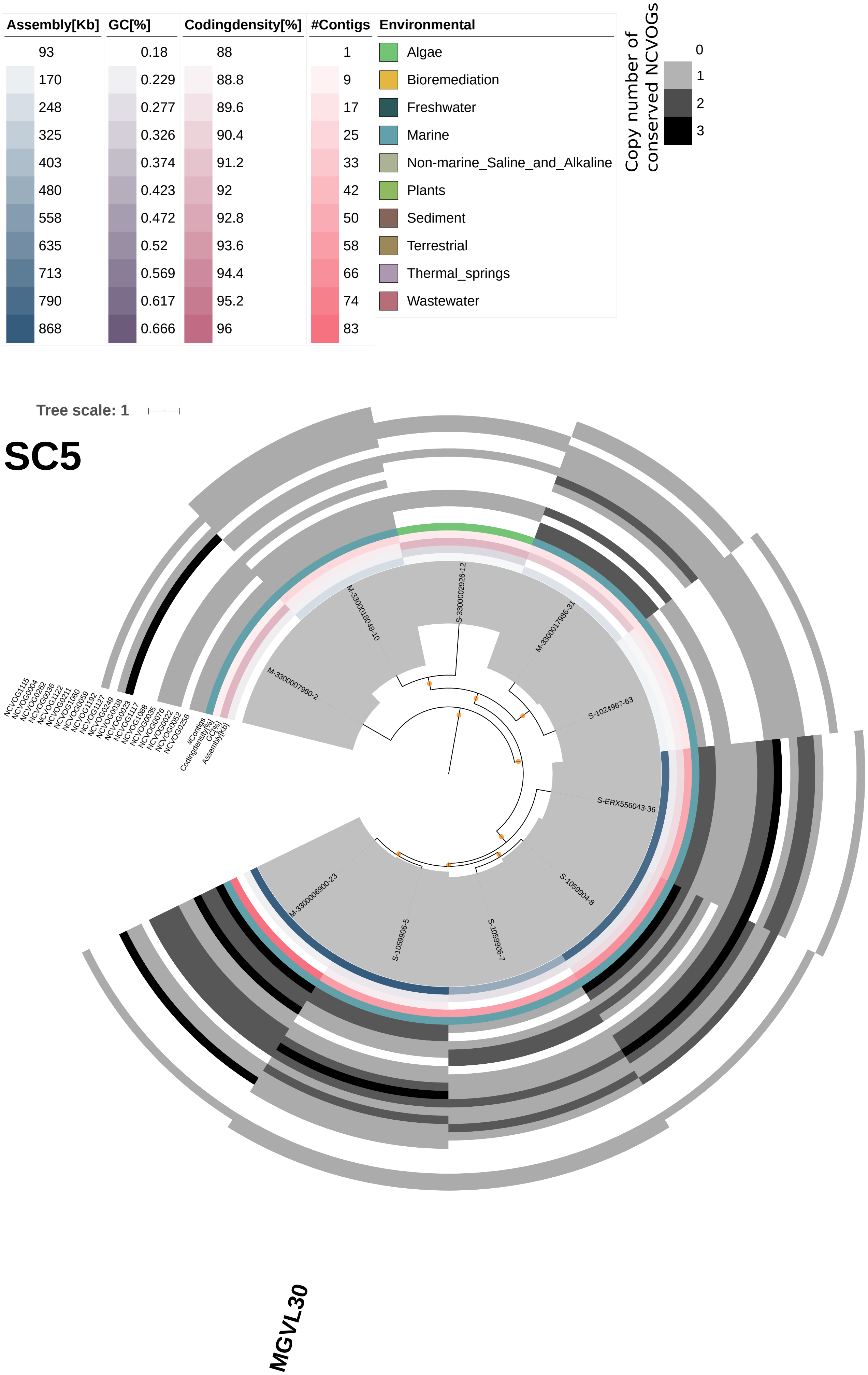

Assembly[Kb]
93
170
248
325
403
480
558
635
713
790
868
GC[%]
0.18
0.229
0.277
0.326
0.374
0.423
0.472
0.52
0.569
0.617
0.666
Codingdensity[%]
88
88.8
89.6
90.4
91.2
92
92.8
93.6
94.4
95.2
96
#Contigs
1
9
17
25
33
42
50
58
66
74
83
Environmental
Algae
Bioremediation
Freshwater
Marine
Non-marine_Saline_and_Alkaline
Plants
Sediment
Terrestrial
Thermal_springs
Wastewater
Copy number of conserved NCVOGs
0
1
2
3
Tree scale: 1
SC5
M-3300007960-2
M-3300018048-10
S-3300002926-12
M-3300017986-31
S-1024967-63
S-ERX556043-36
S-1059904-8
S-1059906-7
S-1059906-5
M-3300006900-23
NCVOG1115
NCVOG0004
NCVOG0262
NCVOG0036
NCVOG1122
NCVOG0211
NCVOG1060
NCVOG0059
NCVOG1192
NCVOG1127
NCVOG0249
NCVOG0038
NCVOG0023
NCVOG1117
NCVOG1088
NCVOG0035
NCVOG0076
NCVOG0022
NCVOG0052
NCVOG0256
#Contigs
Codingdensity[%]
GC[%]
Assembly[Kb]
MGVL30

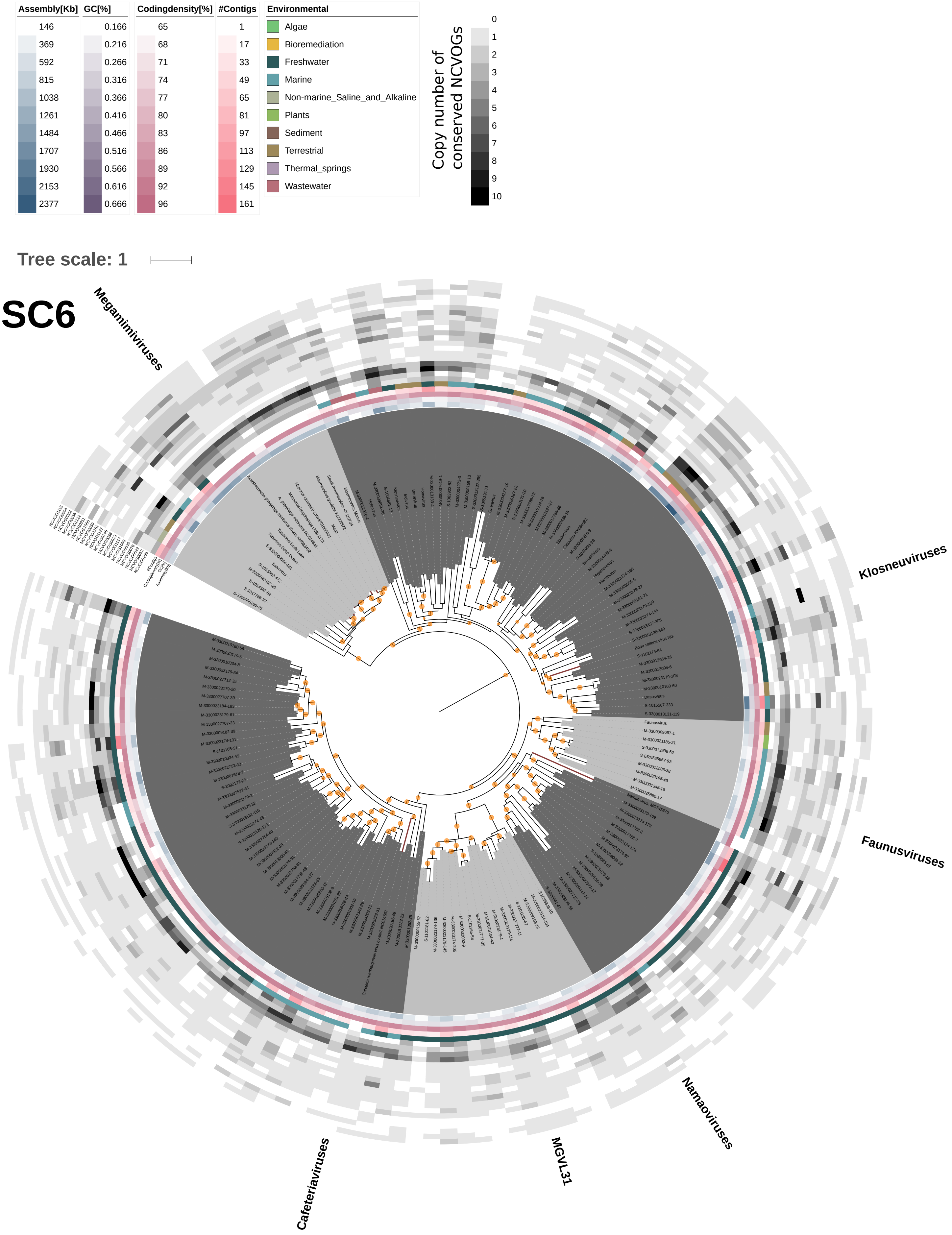

Assembly[Kb]
146
369
592
815
1038
1261
1484
1707
1930
2153
2377
GC[%]
0.166
0.216
0.266
0.316
0.366
0.416
0.466
0.516
0.566
0.616
0.666
Codingdensity[%]
65
68
71
74
77
80
83
86
89
92
96
#Contigs
1
17
33
49
65
81
97
113
129
145
161
Environmental
Algae
Bioremediation
Freshwater
Marine
Non-marine_Saline_and_Alkaline
Plants
Sediment
Terrestrial
Thermal_springs
Wastewater
Copy number of conserved NCVOGs
0
1
2
3
4
5
6
7
8
9
10
Tree scale: 1
SC6
Megamimiviruses
Klosneuviruses
Faunusviruses
Namaoviruses
MGVL31
Cafeteriaviruses

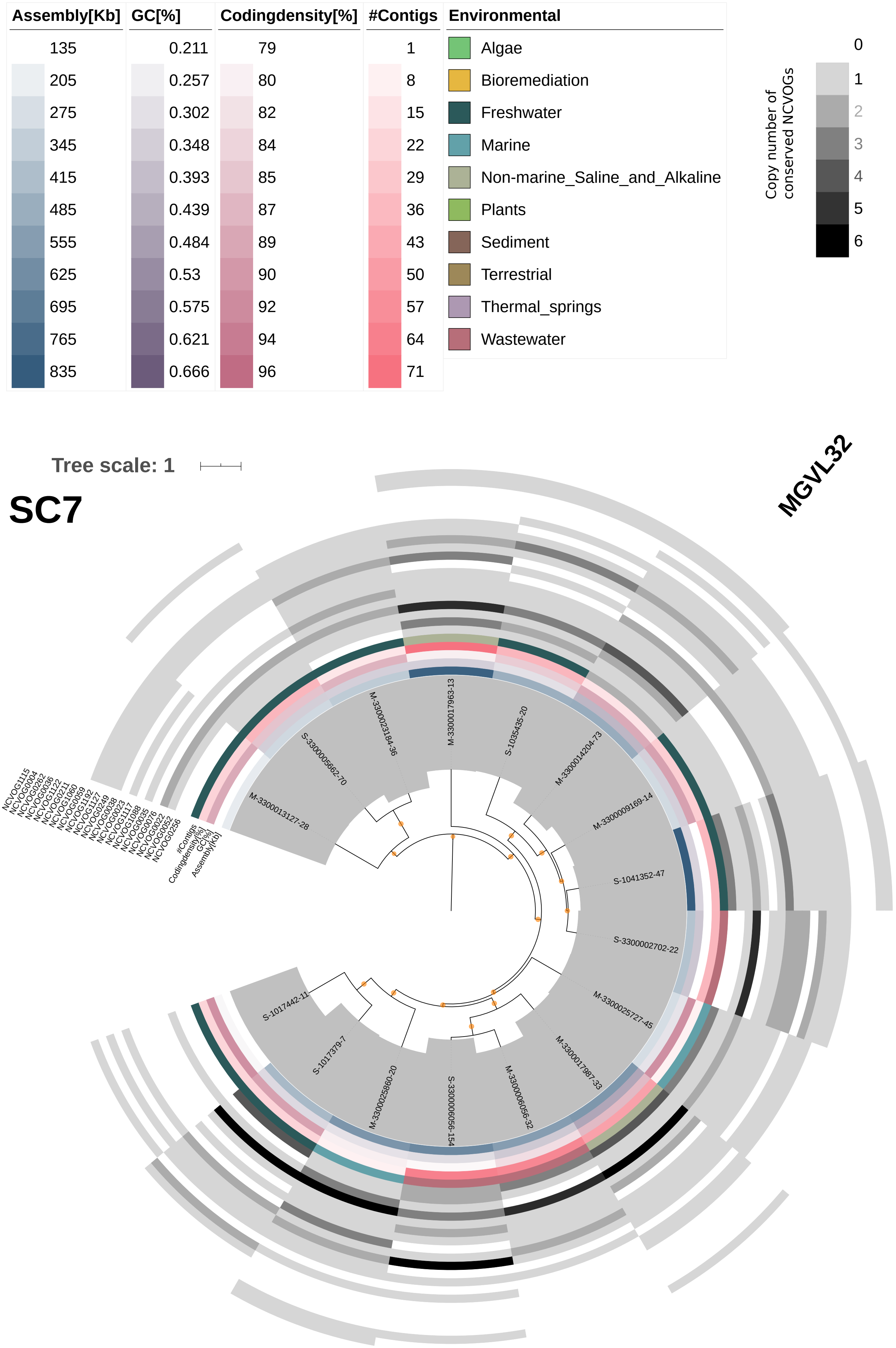

Assembly[Kb]
135
205
275
345
415
485
555
625
695
765
835
GC[%]
0.211
0.257
0.302
0.348
0.393
0.439
0.484
0.53
0.575
0.621
0.666
Codingdensity[%]
79
80
82
84
85
87
89
90
92
94
96
#Contigs
1
8
15
22
29
36
43
50
57
64
71
Environmental
Algae
Bioremediation
Freshwater
Marine
Non-marine_Saline_and_Alkaline
Plants
Sediment
Terrestrial
Thermal_springs
Wastewater
Copy number of conserved NCVOGs
0
1
2
3
4
5
6
Tree scale: 1
SC7
MGVL32
M-3300013127-28
S-3300005662-70
M-3300023184-36
M-3300017963-13
S-1035435-20
M-3300014204-73
M-3300009169-14
S-1041352-47
S-3300002702-22
M-3300025727-45
M-3300017987-33
M-3300006056-32
S-3300006056-154
M-3300025860-20
S-1017379-7
S-1017442-11
NCVOG1115
NCVOG0004
NCVOG0262
NCVOG0036
NCVOG1122
NCVOG0211
NCVOG1060
NCVOG0059
NCVOG1192
NCVOG1127
NCVOG0249
NCVOG0038
NCVOG0023
NCVOG1117
NCVOG1088
NCVOG0035
NCVOG0076
NCVOG0022
NCVOG0052
NCVOG0256
#Contigs
Codingdensity[%]
GC[%]
Assembly[Kb]

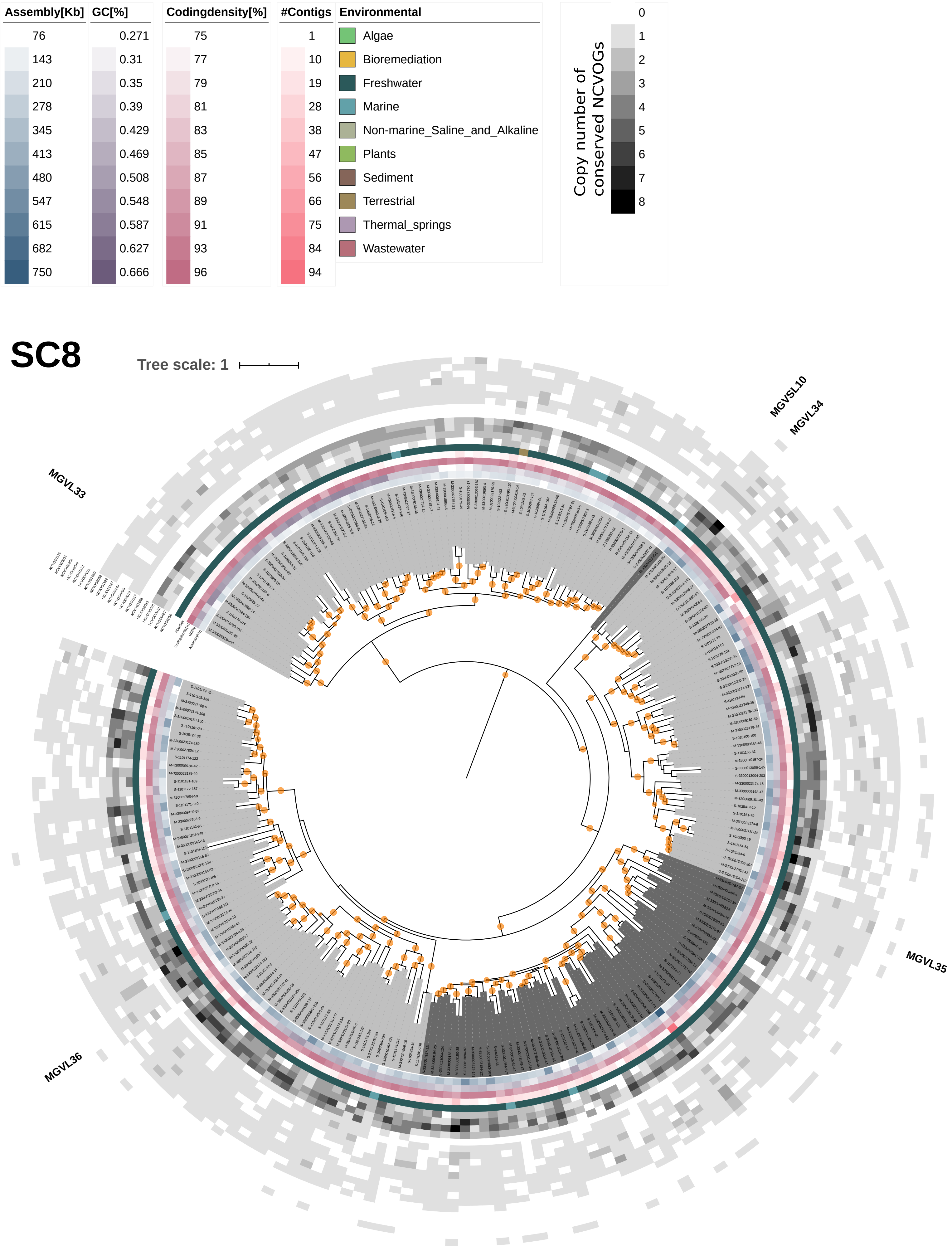
Assembly[Kb]
76
143
210
278
345
413
480
547
615
682
750
GC[%]
0.271
0.31
0.35
0.39
0.429
0.469
0.508
0.548
0.587
0.627
0.666
Codingdensity[%]
75
77
79
81
83
85
87
89
91
93
96
#Contigs
1
10
19
28
38
47
56
66
75
84
94
Environmental
Algae
Bioremediation
Freshwater
Marine
Non-marine_Saline_and_Alkaline
Plants
Sediment
Terrestrial
Thermal_springs
Wastewater
Copy number of conserved NCVOGs
0
1
2
3
4
5
6
7
8
SC8
Tree scale: 1
MGVSL10
MGVL34
MGVL33
MGVL35
MGVL36

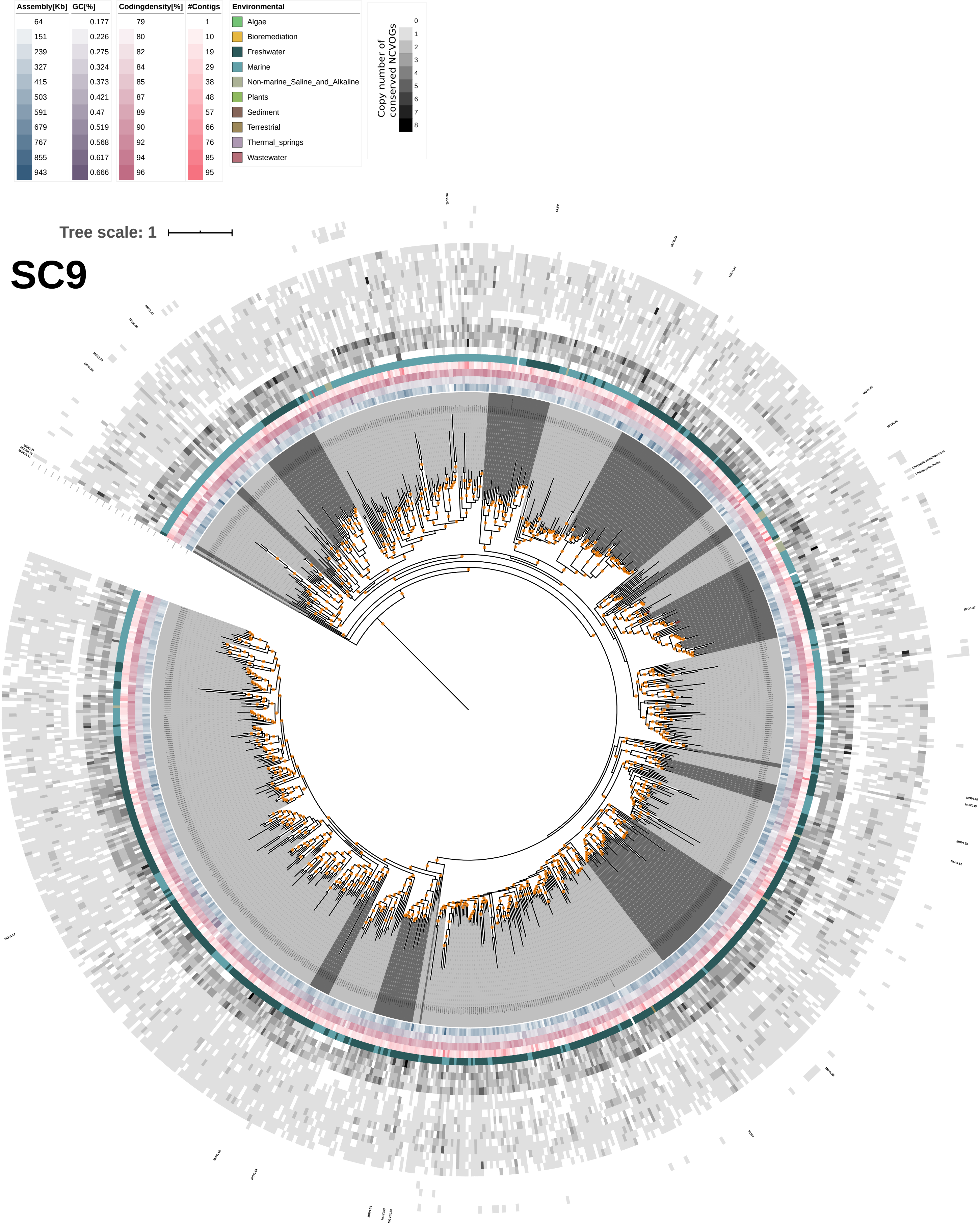
Assembly[Kb]
64
151
239
327
415
503
591
679
767
855
943
GC[%]
0.177
0.226
0.275
0.324
0.373
0.421
0.47
0.519
0.568
0.617
0.666
Codingdensity[%]
79
80
82
84
85
87
89
90
92
94
96
#Contigs
1
10
19
29
38
48
57
66
76
85
95
Environmental
Algae
Bioremediation
Freshwater
Marine
Non-marine_Saline_and_Alkaline
Plants
Sediment
Terrestrial
Thermal_springs
Wastewater
Copy number of conserved NCVOGs
0
1
2
3
4
5
6
7
8
Tree scale: 1
SC9

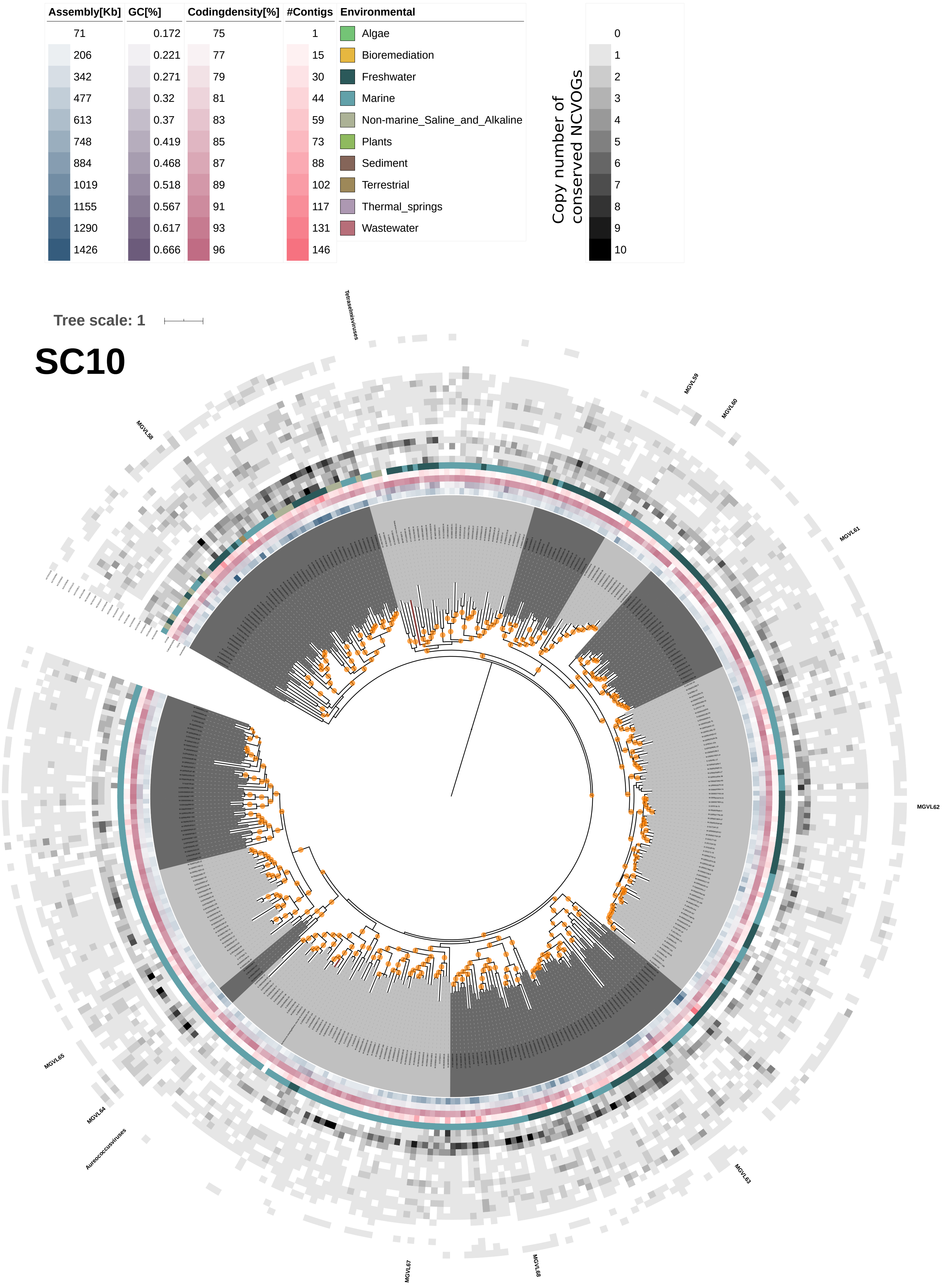
Assembly[Kb]
71
206
342
477
613
748
884
1019
1155
1290
1426
GC[%]
0.172
0.221
0.271
0.32
0.37
0.419
0.468
0.518
0.567
0.617
0.666
Codingdensity[%]
75
77
79
81
83
85
87
89
91
93
96
#Contigs
1
15
30
44
59
73
88
102
117
131
146
Environmental
Algae
Bioremediation
Freshwater
Marine
Non-marine_Saline_and_Alkaline
Plants
Sediment
Terrestrial
Thermal_springs
Wastewater
Copy number of conserved NCVOGs
0
1
2
3
4
5
6
7
8
9
10
Tree scale: 1
SC10
Tetraselmisviruses
MGVL58
MGVL59
MGVL60
MGVL61
MGVL62
MGVL63
MGVL64
MGVL65
MGVL66
MGVL67
MGVL68
Aureococcusviruses
